# Transcriptome Profiling of the Intoxication Response of *Tenebrio molitor* Larvae to *Bacillus thuringiensis* Cry3Aa Protoxin

**DOI:** 10.1371/journal.pone.0034624

**Published:** 2012-04-25

**Authors:** Brenda Oppert, Scot E. Dowd, Pascal Bouffard, Lewyn Li, Ana Conesa, Marcé D. Lorenzen, Michelle Toutges, Jeremy Marshall, Diana L. Huestis, Jeff Fabrick, Cris Oppert, Juan Luis Jurat-Fuentes

**Affiliations:** 1 USDA Agricultural Research Service, Center for Grain and Animal Health Research, Manhattan, Kansas, United States of America; 2 Molecular Research LP, Shallowater, Texas, United States of America; 3 454 Life Sciences, a Roche Company, Branford, Connecticut, United States of America; 4 Bioinformatics and Genomics Department, Centro de Investigación Príncipe Felipe, Valencia, Spain; 5 Department of Entomology, North Carolina State University, Raleigh, North Carolina, United States of America; 6 Department of Entomology, Kansas State University, Manhattan, Kansas, United States of America; 7 USDA ARS U.S. Arid Land Agricultural Research Center, Maricopa, Arizona, United States of America; 8 Department of Entomology and Plant Pathology, University of Tennessee, Knoxville, Tennessee, United States of America; U. Kentucky, United States of America

## Abstract

*Bacillus thuringiensis* (Bt) crystal (Cry) proteins are effective against a select number of insect pests, but improvements are needed to increase efficacy and decrease time to mortality for coleopteran pests. To gain insight into the Bt intoxication process in Coleoptera, we performed RNA-Seq on cDNA generated from the guts of *Tenebrio molitor* larvae that consumed either a control diet or a diet containing Cry3Aa protoxin. Approximately 134,090 and 124,287 sequence reads from the control and Cry3Aa-treated groups were assembled into 1,318 and 1,140 contigs, respectively. Enrichment analyses indicated that functions associated with mitochondrial respiration, signalling, maintenance of cell structure, membrane integrity, protein recycling/synthesis, and glycosyl hydrolases were significantly increased in Cry3Aa-treated larvae, whereas functions associated with many metabolic processes were reduced, especially glycolysis, tricarboxylic acid cycle, and fatty acid synthesis. Microarray analysis was used to evaluate temporal changes in gene expression after 6, 12 or 24 h of Cry3Aa exposure. Overall, microarray analysis indicated that transcripts related to allergens, chitin-binding proteins, glycosyl hydrolases, and tubulins were induced, and those related to immunity and metabolism were repressed in Cry3Aa-intoxicated larvae. The 24 h microarray data validated most of the RNA-Seq data. Of the three intoxication intervals, larvae demonstrated more differential expression of transcripts after 12 h exposure to Cry3Aa. Gene expression examined by three different methods in control vs. Cry3Aa-treated larvae at the 24 h time point indicated that transcripts encoding proteins with chitin-binding domain 3 were the most differentially expressed in Cry3Aa-intoxicated larvae. Overall, the data suggest that *T. molitor* larvae mount a complex response to Cry3Aa during the initial 24 h of intoxication. Data from this study represent the largest genetic sequence dataset for *T. molitor* to date. Furthermore, the methods in this study are useful for comparative analyses in organisms lacking a sequenced genome.

## Introduction

The Gram-positive bacterium *Bacillus thuringiensis* (Bt) produces insecticidal crystal (Cry) toxins that represent the most successful and widely used biopesticides to date. Because Bt toxins offer excellent control of target pests with minimal environmental impact, transgenic crops producing Cry toxins have been developed and widely adopted [1], [2]. Cry toxins are classified according to their amino-acid sequence identity [3], which contributes to specificity in the target pest(s) [4]. For example, Cry1A toxins affect primarily lepidopterans, whereas Cry3A toxins target some coleopterans.

While research is ongoing to unravel the complex mechanism of how Bt toxins kill insects, it is generally accepted that gut proteases in a susceptible insect contribute to the processing of Cry protein inclusions to active toxins that traverse the peritrophic matrix and bind to receptors on the brush border apical surface of gut cells. In Lepidoptera, two models have been proposed to explain how Cry toxins kill enterocytes. According to the pore formation model, binding of Cry toxin monomers to a cadherin-like protein in the brush border membrane results in further toxin proteolysis, toxin oligomerization, binding to secondary receptors, and insertion of the toxin oligomer into the cell membrane to create pores that lead to osmotic cell death [5]. Alternatively, the signal transduction model proposes that binding of Cry toxin monomers to cadherin activates intracellular cell death mechanisms [6]. Common to both models is Cry toxin interaction with a membrane cadherin. We previously reported that binding of Cry3Aa to a membrane cadherin in *T. molitor* larvae is critical for toxicity [7], demonstrating that cadherins are conserved Cry toxin receptors in both lepidopterans and coleopterans.

Despite extensive characterization of the Cry intoxication process in insects, studies of Bt mode of action at the whole-organism level are restricted to those from the nematode *Caenorhabditis elegans*, mainly due to the availability of a well-defined genetic system. Nematode studies have incorporated gene expression analysis, genetic screens, mapping, and RNA interference to demonstrate various pathways involved in Cry toxicity and defensive host responses. Nematodes resistant to Cry5B have alterations in expression of p38 mitogen-activated protein kinase (MAPK), unfolded protein response (UPR), and DAF-2 insulin/IGF1 signalling pathways [8], [9], [10]. Nematodes resistant to Cry21A have an activated hypoxia response pathway [11]. A genome-wide RNAi screen found 106 genes involved in cellular defence against Cry5A, in particular the JNK MAPK pathway [12]. Although these studies with Cry-resistant nematode populations represent the first attempts to understand Bt intoxication in whole organisms, relevance of the data to insects is unknown.

Studies of host response to insecticidal Cry proteins are incomplete due to the lack of genetic information in Bt-susceptible insects. Preliminary studies have utilized partial cDNA microarrays to monitor gene expression patterns during Bt intoxication [13], [14]. The majority of differential gene expression in *Choristoneura fumiferana* and *Manduca sexta* larvae exposed to sublethal doses of Cry1Ab protoxin occurred between two and five hours post intoxication, with mostly metabolic genes repressed and immune and stress response genes induced [14]. Comparisons of gene expression between Bt-resistant and -susceptible lepidopteran insects also have yielded some insights into Bt intoxication processes [15], [16], [17].

In the case of coleopteran insects, the availability of the sequenced genome of *Tribolium castaneum* has promoted the use of this coleopteran insect as a model for studies of generalized insect development and pest biology [18], [19]. However, *T. castaneum* is insensitive to Bt, requiring extremely high doses for toxicity [20] and therefore has limited value in studying Bt intoxication. In contrast, larvae of the yellow mealworm, *Tenebrio molitor,* are susceptible to the coleopteran-specific toxin Cry3Aa at doses similar to Cry1A toxins in Lepidoptera [20], [21], [22], and this species was used to identify the first Bt toxin-receptor in Coleoptera [7]. However, the largest acquisition of genetic data for this species has previously been through our EST study of digestive proteases in the larval gut [23].

In the present study, we used high-throughput pyrosequencing and transcriptome profiling (RNA-Seq) to examine the effect of Cry3Aa intoxication on the *T. molitor* larval gut transcriptome. From these data, we developed gut-specific custom microarrays for *T. molitor* to evaluate temporal changes in gene expression during Cry3Aa intoxication and validate information from RNA-Seq experiments. We also used qPCR to further study the expression of transcripts encoding proteins with chitin-binding domain 3, which were identified in RNA-Seq and microarrays as the genes with most significantly altered expression during Cry3Aa intoxication. Our data suggest a complex response to Cry3Aa intoxication involving shifts in cellular respiration, metabolic energy, signalling, immune recognition, and membrane restructuring.

## Results

### High-throughput Sequencing, Assembly and Annotation

Our goal was to design an experiment to obtain midgut-specific transcriptomic data from the coleopteran *T. molitor*, which lacks a sequenced genome, and also analyze differential gene expression in response to Cry intoxication. In this experiment, we chose a 24 h *ad libitum* exposure of *T. molitor* larvae to diet containing a dose of Cry3Aa protoxin equivalent to the LC_50_ in two-week bioassays [20]. Mortality was not observed over the 24 h period, but larvae were presumably in the early stages of intoxication.

A single Roche GS-FLX sequencing run generated 258,377 usable reads from two samples of *T. molitor* larval gut mRNA (control and Cry3Aa-treated), with an average read length of 249 bp (data deposited at the NCBI Sequence Read Archive). Sequence reads were grouped into two datasets: 124,287 reads from larvae fed a diet containing 0.1% Cry3Aa protoxin (w/w), and 134,090 from a control group reared on untreated diet. Each dataset was independently assembled (assembly 1), with sequences from the control group assembled into 1,318 contigs (individual contigs from the control group are referred to as “Cont-” followed by the contig number), and sequences from the Cry3Aa-treated group assembled into 1,140 contigs (individual contigs from this group are referred to as “Bt-” followed by the contig number).

### Blast2GO Analysis of Assembly 1

Blast2GO [24] was used to assign putative functional groups in predicted proteins from control and Cry3Aa-treated contig sequences. Sequences were submitted for BLASTx analysis with NCBI’s non-redundant (nr) database, and Gene Ontology (GO) associations [25] were obtained. Among all contigs, 2,121 (86%) were similar (e<10^–5^) to proteins in the nr database. A total of 22,696 and 16,395 GO terms were assigned to control and Cry3Aa-treated contig datasets, respectively. Typical gut-specific functions, such as food digestion and storage, energy production, regulation of mRNA translation, and protection of the integrity of the gut were indicated by GO terms (Tables S1 and S2). Contigs with the highest number of reads in both datasets were associated with cytochrome b/cytochrome c oxidase, NADH dehydrogenase, proteases, and glycosidases (data not shown).

#### Enrichment analyses

**Figure 1:**
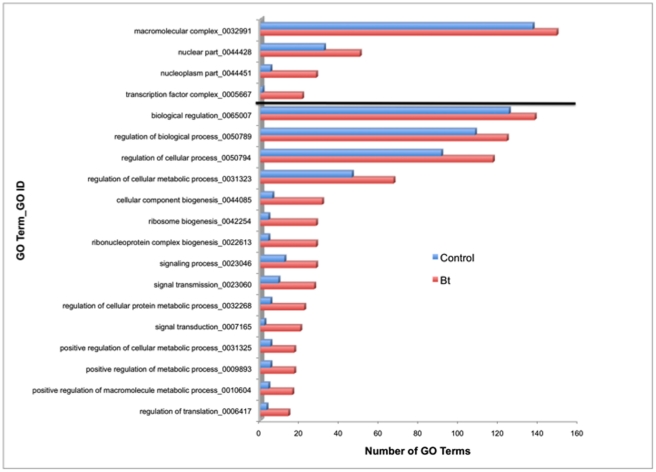
Gene Ontology (GO) terms and numerical identifications (ID) from annotations enriched significantly (Fisher’s Exact Test, p<0.05) in a Blast2GO analysis of transcripts from the gut of *Tenebrio molitor* larvae fed diet containing 0.1% Cry3Aa for 24 h compared to larvae fed control diet, in the categories Cellular Component (above black line) and Biological Process (below black line).

The functional enrichment program currently implemented in Blast2GO applies the Fisher’s Exact Test to annotated sequences without consideration of the number of reads. The results from this statistical analysis (p<0.05) depicted an increase in the number of GO terms in Cellular Components (CC) and Biological Processes (BP) in Cry3Aa-treated larvae (Fig. 1). More specific GO terms in BP indicated increased translation, metabolic processes, and signalling, while those in CC suggested an increase transcription factors and other nuclear components. In this analysis (without associated number of sequence reads), GO terms in the category Molecular Function (MF) were not enriched, and there were no under-represented terms in Cry3Aa-treated larvae.

To improve the enrichment analysis, a custom script was developed to incorporate the length and number of reads associated with each contig in the statistical test, providing a quantitative analysis of the RNA-Seq data. In this analysis, GO categories were identified that contained terms that were significantly (p<0.05) enriched (Table S1) or repressed (Table S2) in Cry3Aa-treated compared to control datasets.

GO terms that were highly enriched in Bt-treated larvae included those associated with transport, cellular respiration, signalling, transcription/translation/protein synthesis, protein recycling, cell structure, and membrane components (Table S1). In particular, highly induced terms associated with transport (proton, sodium ion, mitochondrial) and mitochondrial functions (respiratory chain complex IV, ubiquinone) suggest that maintenance of ionic homeostasis and energy production are challenged in intoxicated larvae. There was an increase in functions associated with the GO term signalling, and annotations of transcripts included polyadenylate-binding protein, G protein-coupled receptors and GTP-binding proteins (including Ras and Rho), kinase (including p38 MAPK), and proteasome components that signal ubiquitination and protein recycling. These data indicated that intoxicated larvae were increasing the production of proteins through an induction of transcription factors and RNA-binding proteins, as well as ribosome and translational events. Maintenance of cell structure and membrane integrity was supported by an increase in cell components, such as tubulin, microtubule, fusome, and subapical complex. Transcripts encoding glycosyl hydrolases (trehalase and α-amylase) also were induced.

In contrast, GO terms that were highly repressed in Cry3Aa-intoxicated larvae included those associated with peptidases, glycolysis, microvilli maintenance, fatty acid catabolism, and antioxidant activity (Table S2). The most significantly repressed GO term was metallopeptidase activity, mostly because of a reduction of transcripts encoding methionine aminopeptidase. Transcripts encoding glycolytic and TCA enzymes (glyceraldehyde 3 phosphate, malate, pyruvate, isocitrate, and 2-oxoglutarate dehydrogenases; pyruvate kinase, enolase, triose phosphate isomerase, aldolase, succinyl-coA synthetase) also were severely repressed in intoxicated larvae. The GO term “border follicle cell migration” and myosin-related terms were underrepresented in intoxicated larvae, largely due to decreased expression of myosin vi, critical to microvilli maintenance [26]. A reduction in terms associated with fatty acid catabolism was associated with a loss of transcripts associated with the peroxisome in intoxicated larvae. Transcripts encoding antioxidant enzymes, including superoxide dismutase, glutathione S transferase, and catalase were also reduced in intoxicated larvae. Other repressed biological processes were associated with DNA modification, cell differentiation, binding, and fucosidase function.

### Microarray Analyses of Assembly 2

In another assembly, raw sequence reads from pyrosequencing of gut mRNA from control and Cry3Aa-treated *T. molitor* larvae were combined into one dataset, retaining information on relative number of reads, and assembled into 25,201 contigs that were used to design oligonucleotide probes for custom microarrays. Total RNA from larvae exposed to Cry3Aa for 6, 12, or 24 h was hybridized to the microarray, and transcript levels from intoxicated larvae at each time-point were compared to unexposed control larvae using pairwise analyses or ANOVA.

#### Pairwise analyses

**Figure 2:**
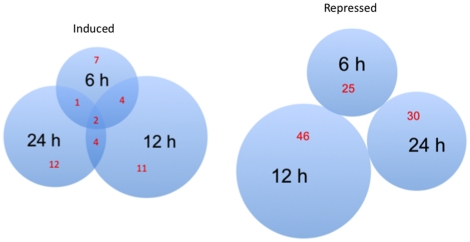
Venn diagrams of the number of genes that were significantly (p<0.05) induced or repressed, as determined by a pairwise analysis of microarray data from *Tenebrio molitor* larvae fed 0.1% Cry3Aa for 6, 12, or 24 h.

In pairwise analyses, a similar number of genes was found to be significantly (p<0.05) induced at 6, 12, and 24 h after Cry3Aa-intoxication, although there was little overlap (Fig. 2, induced). However, there were more genes that were significantly repressed in *T. molitor* larvae exposed to Cry3Aa for 12 h compared to the other time-points, and no overlap (Fig. 2, repressed). Overall, more changes were observed in larvae exposed to Cry3Aa for a 12 h time interval. We speculate that distinct biological events were occurring at each intoxication time point, since few genes were common at multiple intoxication intervals.

In comparing control *T. molitor* larvae to those exposed to Cry3Aa for 6 h, a transcript encoding a putative allergen-like protein (AY327800) was increased in expression more than 8-fold in intoxicated larvae (Table S3). The cDNA for AY327800 in NCBI (no associated publication) was isolated from the microvillar membrane of *T. molitor* larvae and encodes a protein belonging to pfam06757 (insect allergen related repeat, containing a nitrile-specifier protein that has adapted to detoxify the plant glucosinolate-myrosinase system) [27]. Transcripts encoding chitin deacetylase and an ortholog to *D. melanogaster* CG14949, expressed mostly in the midgut/hindgut of larvae and hindgut of adult flies [28], were induced 2-fold in Cry3Aa-intoxicated larvae. Other transcripts were induced <2-fold at 6 h but may have relevance to intoxication. One transcript encoded a cathepsin L-like protein (AY337517), previously described as a major digestive peptidase in *T. molitor* larvae [29]. The increase in this transcript may represent an attempt by larvae to preserve cysteine peptidase activity in the anterior midgut, a critical component of the initial digestion of protein in this insect [30]. As in the enrichment analysis of RNA-Seq data, transcripts related to tubulin were also increased in the 6 h microarray.

In 6 h Cry3Aa-intoxicated *T. molitor* larvae, a transcript encoding melanin-inhibiting protein, a negative regulator of melanin synthesis in *T. molitor* larvae [31], was repressed 1.98-fold compared to control (Table S3). Cellular and other humoral responses in insects, such as melanization, are involved in rapid response to infection or pathogens [32]. Although only slightly repressed, some transcripts may be related to the Bt-intoxication response: synaptic vesicle protein, possibly a neurotransmitter; a receptor for renin (aspartic protease) that upon binding activates MAPK kinases ER1 and ER2 [33]; a serine-threonine protein kinase that also may be involved in signalling; and several transcription/translation-related proteins.

At 12 h post intoxication, the most highly induced transcript encoded a protein with a chitin-binding domain 3 (CBD3) and another lacking annotation (Table S4). One induced transcript was related to a serine protease (DQ356028) that was isolated previously from the anterior midgut of *T. molitor* larvae and was annotated as a protease homolog lacking peptidase functional residues. Protease homologs have been proposed to be involved in detoxification of cereal protease inhibitors [23]. Another induced transcript, glycosyl hydrolase (family 2), was similar to a *T. castaneum* predicted gene (XM_964186) that is highly expressed in the larval gut [19]. Other slightly induced transcripts included dynactin, which is involved in bidirectional intracellular organelle transport by binding to dynein and kinesin II, and tryptophanyl-tRNA synthetase/ligase, and in humans interacts with VE-cadherin to protect endothelial cells against stress [34]. Tubulin and transcription/translation events remained induced in 12 h Cry3Aa-intoxicated larvae.

As previously noted, more transcripts were repressed in 12 h-intoxicated *T. molitor* larvae (Table S4). In particular, one of the more severely repressed transcripts encoded a putative lipase, and another lipid-related transcript was related to phospholipid scramblase. Repressed transcripts encoding nuclear genes included: RBBP4, encoding a nuclear protein integral to transcriptional silencing; Pho, a DNA-binding protein involved in transcription repression of homeotic genes; and “small nuclear ribonucleoprotein at 69D” involved in RNA processing. Repressed immune-related transcripts included: 86 kDa encapsulation protein, serpin peptidase inhibitor 31, peroxiredoxin, and an inhibitor of NFkappaB kinase. Other repressed transcripts encoded putative metabolic enzymes such as xanthine dehydrogenase, chitinase, esterase, serine protease, adenosylhomocysteinase, 6-phosphogluconate dehydrogenase, and thiolase/acetyltransferase. Repressed transcripts encoding a protein with a TBC domain that interacts with GTPases may be involved in signalling. In summary, at 12 h a more complex response to protoxin was observed, in which transcripts related to immunity, metabolism, signalling, and transcription/translation were differentially expressed.

At 24 h post Cry3Aa intoxication, transcripts containing CBD3 remained induced in intoxicated larvae (Table S5). In addition, a transcript encoding the allergen-like protein CG10477, induced in 6 but not 12 h-intoxicated larvae, was induced again at 24 h. Transcripts associated with chitin deacetylase also were induced. Several transcripts encoding thaumatin-like proteins (TC011564 and TC000515) were induced at 24 h. In *T. castaneum*, thaumatin transcripts are highly expressed in the larval gut [18] and may be involved in antimicrobial responses [35]. As observed at 12 h post intoxication, transcripts encoding putative glucosyl hydrolase (β-glucosidase) were also induced at 24 h. Some transcripts that were induced at 6 h were also induced at 24 h, including those encoding additional allergen-like and cathepsin L proteins. At 24 h, there was increased expression of one serine protease transcript (DQ356032, annotated as elastase-like) and decreased expression of another (TC012575, trypsin-like). Consistent at all time-points was the induction of β-tubulin transcripts. Although β-tubulin is often used as a housekeeping gene in gene expression studies, our data indicate this gene would not be a good choice as a normalizer in studies of Cry intoxicated insects. Fewer transcripts were repressed at 24 h than 12 h, but the most severely repressed was one also encoding a CBD3 protein. Other repressed transcripts implicated in immunity were related to TC012575 [36], a dust mite allergen Aca s 13 that may be involved in lipid binding [37], and B-cell receptor-associated protein 31-like that may transport membrane proteins from the endoplasmic reticulum to the Golgi [38] and may be involved in CASP8-mediated apoptosis [39].

Comparisons of differential expression between the 24 h intoxicated larval response in the microarray and RNA-Seq experiment were mostly in agreement (Table S5). In particular, the different methods validated differential expression of transcripts encoding putative chitin-interacting proteins (binding, deacetylase), allergens, thaumatins, glucosidases, and serine proteases. Differences were observed with respect to directional (increased or repressed) expression for melanin-inhibiting protein, receptor expression enhancing protein, allergen Aca s 13, and a few lacking annotation. One explanation for the differences may be that larvae in the RNA-Seq experiment were exposed to Cry3Aa *ad libitum*, whereas in the microarray protoxin exposure was more tightly regulated. Without a sequenced genome, it is also plausible that there may be errors in array probe design. These data may be improved with larger RNA-Seq datasets consisting of longer reads, and increased biological replicates in the microarray.

#### Principle component analysis

**Figure 3:**
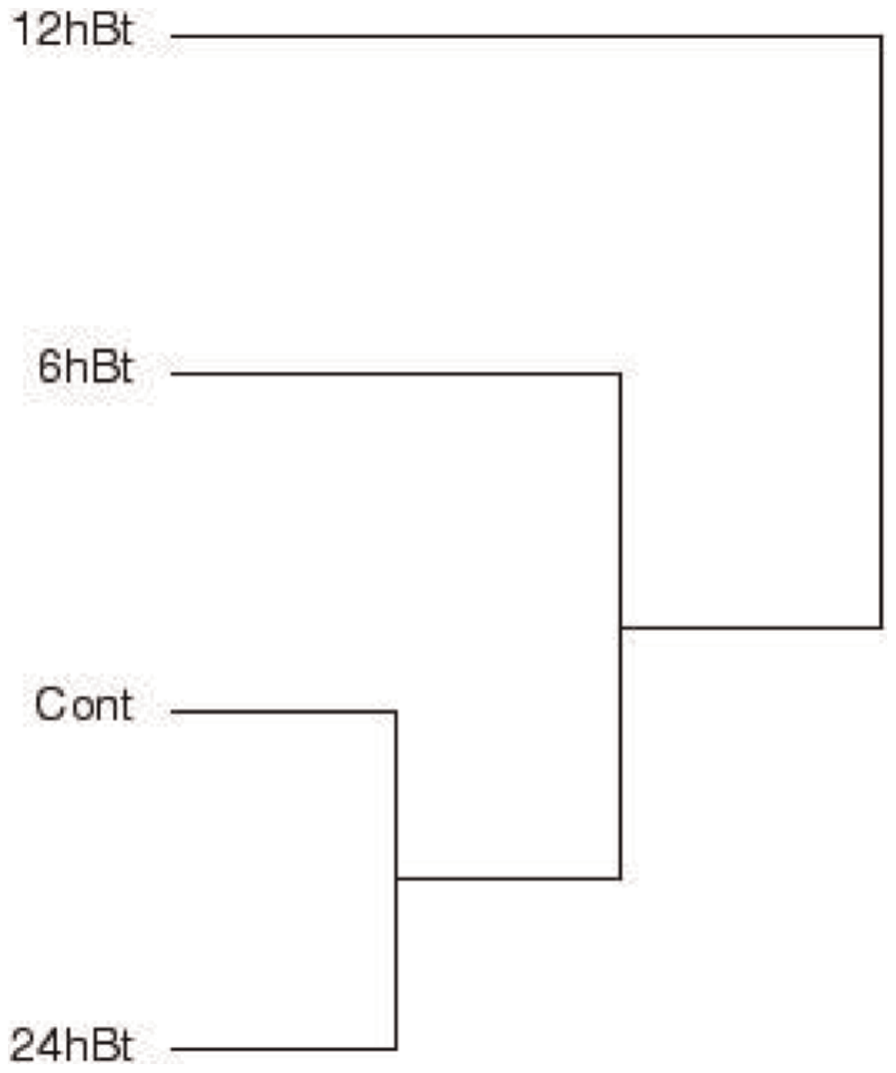
Principle component analysis (Manhattan distance, Average linkage) of datasets from control, 6 h, 12 h, or 24 h Cry3Aa-intoxicated (Bt) *Tenebrio molitor* larvae.

Principle Component Analysis (PCA) is a measure of potentially correlated variables (i.e., gene expression levels as determined by signal intensities) grouped into uncorrelated variables, called principle components, by a mathematical transformation. With PCA analysis of our unfiltered microarray data, the variance in gene expression between the four different treatments suggested that gene expression in control was more similar to 24 h-intoxicated larvae, perhaps an indication that some larvae may be initiating gut recovery processes in an attempt to maintain homeostasis, whereas expression in 12 h-intoxicated larvae was most different among all treatments (Fig. 3). Thus, in agreement with results from pairwise analysis, PCA data also indicated that differences in gene expression in *T. molitor* larvae were more significant after 12 h Cry3Aa protoxin exposure.

#### ANOVA analysis of control and all intoxication time-points

**Figure 4:**
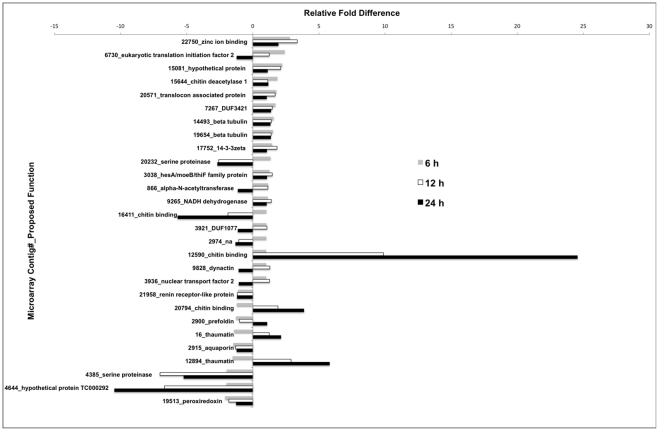
ANOVA of significant (p<0.05) differences in gene expression patterns of transcripts from *Tenebrio molitor* larvae fed 0.1% Cry3Aa for 6, 12, or 24 h compared to those fed control diet, hybridized to a custom microarray and expressed as relative fold difference. Microarray contig# and putative protein are on the left, based on tBLASTx of NCBI nr; na, not available due to lack of homology or annotation.

As in the pairwise analysis, differential expression of *T. molitor* transcripts associated with chitin-binding and metabolism were found in the ANOVA analysis of all exposure times (Fig. 4). In fact, expression of contig_12590 (encoding CBD3) was induced at all time-points in Cry3Aa-intoxication, especially at 12 and 24 h. This transcript is related to *Drosophila* CG4367, which is highly expressed in the larval midgut and hindgut of *D. melanogaster* [28] and is induced in response to gram-negative bacterial infection [40]. Most of the transcripts that were induced over all time-points were similar or identical to those in the pairwise comparisons. Exceptions included transcripts encoding a translocon associated protein potentially binding calcium to the membrane of the endoplasmic reticulum (ER) to retain ER resident proteins, several proteins associated with mRNA translation (eIF2 and hesA/moeB/thiF family), and 14-3-3 zeta with diverse functions in signalling pathways. Many transcripts were repressed in the pairwise comparison and in the ANOVA analysis. Exceptions found only in the ANOVA analysis included those encoding peroxiredoxin, a bifunctional enzyme with glutathione peroxidase and phospholipase A2 activities and suggested to be involved in the immune response in *T. castaneum* [36], and a hypothetical protein (related to TC000292) with unknown function.

#### Expression patterns of putative Bt-toxin receptors

**Figure 5:**
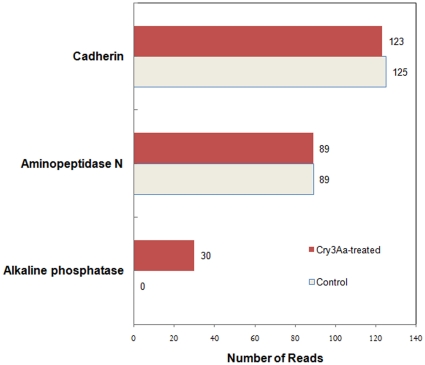
RNA-Seq expression of transcripts encoding putative Bt toxin receptors in control and Cry3Aa-treated *Tenebrio molitor* larvae, comparing the number of reads of transcripts encoding proteins related to cadherin, aminopeptidase N, and alkaline phosphatase in each treatment group.

Of the putative Bt toxin receptors identified from different insect orders, the three more characterized receptors are cadherin, aminopeptidase N, and alkaline phosphatase [41]. We compared expression levels of contigs related to these putative toxin receptors in RNA-Seq by examining the number of reads (Fig. 5). Contigs related to aminopeptidase N or cadherin had similar numbers of reads in control or Cry3Aa-intoxicated larvae. In contrast, transcripts related to alkaline phosphatase were found only in intoxicated larvae. Expression patterns of transcripts related to putative Bt toxin receptors in the microarrays were not statistically significant (p<0.05; data not shown).

### Expression Analyses of Chitin-binding Domain 3 Transcripts

**Figure 6:**
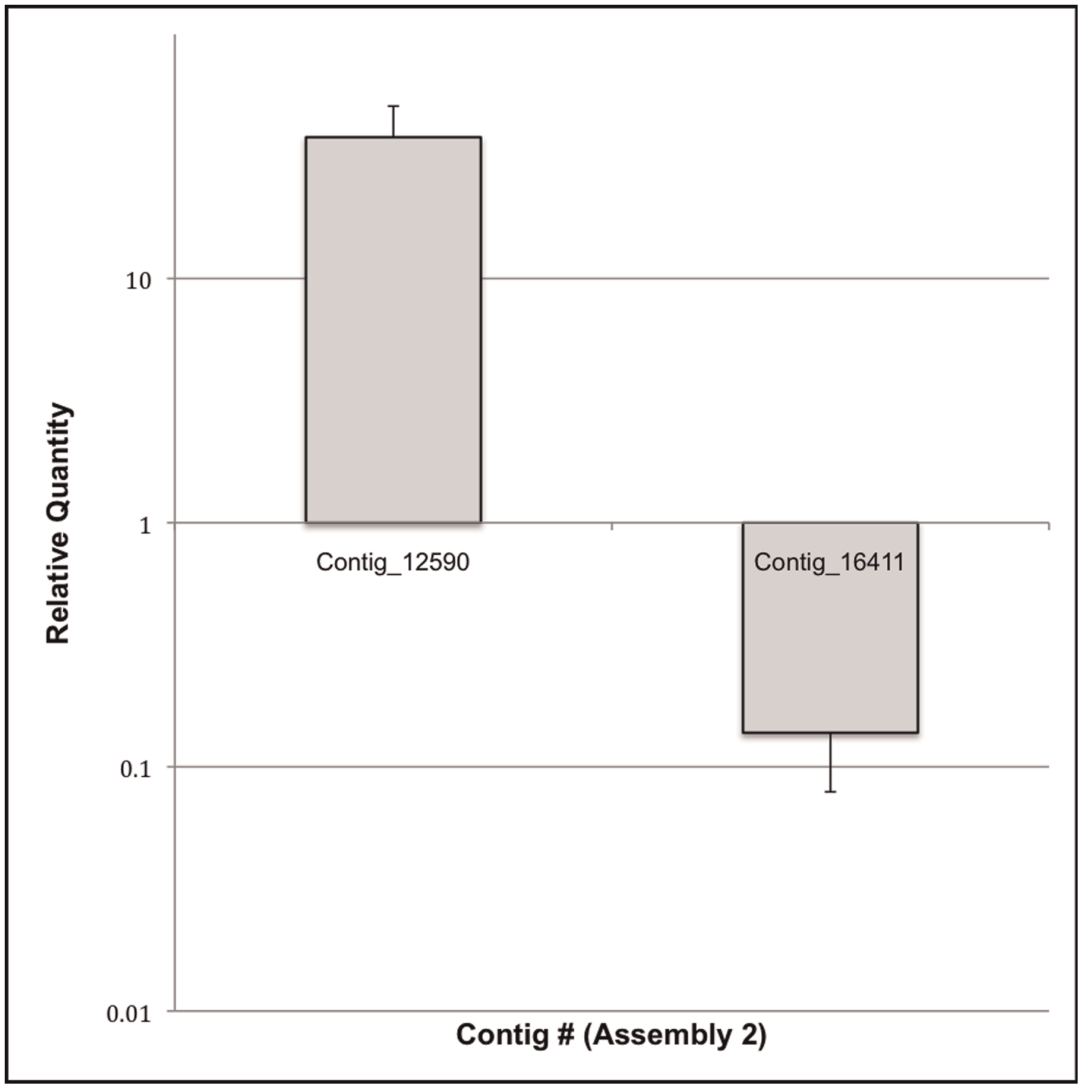
Relative expression of *Tenebrio molitor* gut transcripts encoding proteins containing chitin-binding domain 3 in Cry3Aa-intoxicated larvae compared to control.

RNA-Seq and microarray analyses indicated that transcripts encoding proteins with CBD3 were the most differentially expressed in *T. molitor* larvae exposed to Cry3Aa for 24 h (Table S5). To further examine the expression of CBD3 transcripts, we used qPCR to verify that transcripts corresponding to Contig_12590 were enriched, whereas transcripts corresponding to Contig_16411 were repressed in 24 h Cry3Aa-treated larvae (Fig. 6). In fact, qPCR indicated that the relative abundance of Contig_12590 transcripts was 37.9-fold more in Cry3Aa-treated than control larvae, more than the relative abundance in RNA-Seq (1.62-fold) but similar to micorarray (24.6-fold). Similarly, qPCR demonstrated that the relative abundance of Contig_16411 was –7.2-fold decreased in Cry3Aa-treated compared to control larvae, more than RNA-Seq (–1.49-fold) but similar to microarray (–5.68-fold).

When the microarray oligo sequences were compared to protein databases (BLASTx, NCBInr), those from Contig_12590 were more closely related to *T. castaneum* Tc016344/6, whereas Contig_16411 oligos related more to Tc016349 (data not shown). We found nine sequences in the predicted genome of *T. castaneum* with CBD3, seven of which are predicted to be in tandem on chromosome 7 (Fig. S1). All but one of CBD3 sequences (TcCBD3_16345) contain a predicted signal peptide, and six of the seven chromosome 7 CBD3 transcripts were found to be highly expressed in the gut of *T. castaneum* larvae fed a normal rearing diet by a previous microarray analysis [19]. In a phylogenetic analysis of predicted CBD3 proteins from *T. molitor* and *T. castaneum*, contig sequences from *T. molitor* were more closely related to each other, and both were more similar to TcCBD3_16349, whereas TcCBD3_16344/6 were more distantly related (Fig. S2).

## Discussion

In this study, we report the combined use of transcriptome profiling and microarray analysis to characterize the response of coleopteran larvae to Cry toxins. Collectively, the data portray a complex response to Cry3Aa intoxication in the *T. molitor* larval gut. Cry1Ab intoxication of *C. fumiferana* and *M. sexta* induced the differential expression of genes related to metabolism and immunity [14]. Similarly, the response of *D. melanogaster* to gram-negative bacteria was the induction of immune response genes and repression of genes associated with digestion [40]. While our data is in agreement with the down-regulation of digestion, Cry3Aa intoxication of *T. molitor* larvae resulted in a varied immune response, but mostly we observed a suppression of immune-related functions in the first 24 h following intoxication. We were able to determine through GO term analysis that Cry3Aa-intoxication resulted in an induction of genes involved in mitochondrial electron transport, signalling, carbohydrate metabolism, membrane components, cell structure, and allergens, while those encoding metabolic enzymes associated with proteolysis, glycolysis, TCA, and fatty acid metabolism were repressed. Similarly, enzymes involved in cellulose degradation were down-regulated in *Diabrotica virgifera* larvae exposed corn roots expressing Cry3Bb [42]. In insects undergoing intoxication, cessation of feeding may result in the down-regulation of digestive-associated processes and up-regulation of energy production via cellular respiration.

The chronology of Cry intoxication is highly variable depending on the target insect, with some lepidopteran larvae demonstrating clear symptoms (cessation of feeding and/or paralysis) within hours after intoxication with Cry1A toxins. In contrast, *T. molitor* larvae can survive Cry3Aa intoxication for weeks without obvious signs of paralysis. Therefore, while *C. fumiferana* and *M. sexta* larvae exposed to sublethal doses of Cry1Ab protoxin displayed the most significant differential gene expression between two and five hours post intoxication [14], we chose to expose *T. molitor* larvae to a lethal dose of Cry3Aa protoxin for 24 h before performing RNA-Seq to ensure all larvae had sufficient exposure to the protoxin, and to ensure mRNA was harvested well in advance of the onset of mortality [20]. The same incubation period was used to characterize Cry3Bb intoxication in another coleopteran pest, *D. virgifera* [42]. Data from the RNA-Seq experiment provided sequence information for the design of microarrays, as well as a snapshot of transcription 24 h post intoxication. Taken together, RNA-Seq (24 h) coupled with microarray analysis (6, 12 and 24 h) provided insight into the intoxication process at three distinct time-points, as well as validation of the methods at 24 h. Interestingly, PCA analysis of the microarray data indicated that differences in 24 h-intoxicated larvae were more similar to control, suggesting that recovery of some genes to levels of normal homeostasis may be occurring by 24 h post intoxication. Based on the number of differentially-expressed genes in the present study, we estimate that intoxication periods between 6 and 12 h are optimal to study Cry3Aa intoxication in *T. molitor.*

Different assemblies of the pyrosequencing data were used in our analyses. With the earlier version of the Roche GS *de novo* Assembler (assembly 1), we had fewer but larger contigs, and comparatively fewer errors in assembly (data not shown). However, with the Seqman NGEN assembly (assembly 2), we retained transcripts that were discarded in the first assembly that may have been important to Cry toxicity, but we found more assembly errors. For example, only one thaumatin-related contig (Bt-01725) was found in the first assembly, while two additional thaumatin-related contigs (16 and 12894) were detected in the second assembly.

Manual inspection of the assemblies also may provide important information. In our case, this was particularly true in a search for transcripts encoding immune-related proteins. For example, transcripts encoding tenecin, a protein related to the insect defensin family with strong activity against Gram-positive bacteria [43], were increased 1.9-fold in the Cry3Aa-treated dataset, but were not found in the Blast2GO statistical analyses. However, transcripts encoding a putative calreticulin were found only in control larvae. Calreticulin is also an immune-related protein found in many organisms, and in the mosquito midgut microvilli it interacts with the P25 surface protein from *Plasmodium vivax* [44]. Transcripts encoding apolipophorin 3, which provides an antibacterial response and other immune-related functions in insects [45], were found only in Cry3Aa-treated larvae.

Microarray analysis revealed that transcripts encoding proteins with CBD3 displayed the most differential expression in Cry3Aa-intoxicated larvae after 24 h intoxication. These proteins are related to CBD3 proteins in *T. castaneum* and *Drosophila* CG4367 that are highly expressed in the gut [19], [28]. CG4367 is induced by Gram-negative bacteria early (4 h) post infection in *D. melanogaster* larvae [40]. CBD3 proteins are highly conserved in plants and animals, but their function is unknown. We confirmed the expression patterns of two of the *T. molitor* CBD3 transcripts in control and Cry3Aa-intoxicated larvae by qPCR, and we determined that a gene expansion in *T. castaneum* has resulted in seven orthologous sequences, encoding highly related but phylogenetically-distinct proteins. The relevance of CBD3 proteins to Cry intoxication and bacterial infection in general appears to be an important area for future studies.

Both RNA-Seq and microarray analysis revealed induction of chitin-specific enzymes, such as chitin deacetylase and chitin synthase, in Cry3Aa-intoxicated larvae. In addition, enrichment analysis of the RNA-Seq data indicated that cellular components related to the maintenance of cell structure were induced upon intoxication. This is in agreement with data from Cry3Bb-intoxtication of *D. virgifera* larvae, where the expression of genes involved in the synthesis of structural proteins is increased upon intoxication [42].

Studies on the mode of action of Cry toxins have been mostly limited to the identification of putative toxin receptors. Previously, we determined that Cry3Aa interacts with a midgut membrane cadherin in *T. molitor* larvae, TmCad1, resulting in toxin oligomerization, and this interaction is crucial to toxicity [7]. In our RNA-Seq analysis (24 h post intoxication), we found that TmCad1 expression was not affected by Cry3Aa-intoxication. However, transcripts associated with alkaline phosphatase, a putative Cry1Ac receptor in Lepidoptera [46], were found only in Cry3Aa-intoxicated larvae in the RNA-Seq analysis. Increased expression of alkaline phosphatase transcripts was also reported in Cry1Ab-resistant *O. nubilalis* [15]. While we found no differences in expression of transcripts encoding the lepidopteran Cry1A receptor alanyl aminopeptidase, methionine aminopeptidase was statistically the most severely repressed transcript in the enrichment analysis, reminiscent of observations in Cry1Ac-resistant lepidopteran larvae [47]. ADAM metalloproteases are another group of putative Cry3Aa toxin receptors in Coleoptera [48]. In our study, an ADAM 17-like gene (microarray contig_2455) was repressed 2.4-fold 12 h after intoxication (data not shown) but was not significant at p<0.05. In examining expression of putative toxin receptors, we are cognizant that changes in transcription levels may not indicate a direct involvement in intoxication (e.g. the most common disruption in toxin binding has been attributed to mutation [49]).

Our results suggest that insect allergens are involved in the response to Cry intoxication. An increased expression of insect allergen-related transcripts was correlated to Cry3Aa-intoxication in both RNA-Seq data and microarray analyses. For example, microarray analysis indicated increased expression of a putative allergen-like protein (microarray contig_18860/AY327800) at both 6 and 24 h post intoxication. RNA-Seq corroborated this finding, and also revealed that transcripts from another allergen, a possible ortholog of MPA2 allergen, were more abundant in Bt-intoxicated compared to control larvae. This allergen has a MD-2-related lipid-recognition domain apparently important for pathogen recognition [50]. More is known about the allergenic response invoked by these proteins in humans than their role in the insect gut.

Analysis of RNA-Seq data revealed enrichment of transcripts associated with signalling in Cry3Aa-treated larvae, including G protein-coupled receptors and MAP kinases. Data from both nematodes and insects supports the involvement of intracellular signalling in Bt mode of action. For example, p21-activated kinase (PAK) intracellular pathways have been implicated in Cry intoxication and gut defensive response in *Caenorhabditis elegans* [8] and *M. sexta* [6]. Signalling processes regulated by PAK proteins are associated with many cellular events, including regulation of the cytoskeleton, cell polarization, control of MAPK signalling cascades, and apoptosis. In fact, a recent report suggests that activation of the MAPK pathway also occurs in Lepidoptera and Diptera in response to Cry1Ab and Cry11Aa intoxication, respectively [51]. This is in agreement with our data, which suggest that coleopterans may respond to Cry3Aa intoxication by invoking the MAPK pathway.

Previous analyses on differential gene expression in response to sublethal Cry intoxication have mostly identified genes involved in catabolism and stress response [13], [14], [42]. Using a combination of functional genomic techniques, we have been able to detect and identify the differential expression of additional genes involved in Cry intoxication. Our work supports the use of high-throughput sequencing as a valuable tool to characterize the Cry intoxication process. Together with our data, the currently available information describes commonalities in the response to Cry intoxication in diverse insect models, including reduced catabolism and activation of immune-related pathways.

We conclude with a retrospect on Bt research. Elucidation of the steps involved in the mode of action of Cry toxins in insects has been an ongoing vigorous research area for almost 30 years. Some research has been more amenable to determining specific genetic alterations that result in decreased toxin susceptibility, while others have addressed specific aspects of intoxication. In contrast to lepidopteran models, information on the genes involved in pathogenesis and response to Cry intoxication in coleopteran larvae is limited. In this study, we demonstrated the combined use of transcriptome profiling and microarray analyses to identify genes differentially expressed in response to Cry3Aa intoxication of *T. molitor* larvae. The differential expression of genes identified in our work may be specific to Cry3Aa pathogenesis or be more generally involved in the gut response to infection. Nonetheless, our study demonstrates that combined use of high-throughput sequencing and other transcriptomic approaches can provide more comprehensive information on the Cry intoxication process in insects lacking sequenced genomes. The current working models on the mode of action of Bt toxins in different insects will be improved with additional sequencing and genetic resources.

## Materials and Methods

### Preparation of Samples for Pyrosequencing

*T. molitor* larvae were taken from the laboratory colony when more than 35 days old (4–7 mg) and were placed in groups of 50 on control diet (85% wheat germ, 10% wheat flour, 5% brewers yeast) or the same diet containing 0.1% w/w Cry3Aa protoxin (Bt *tenebrionis*) as described elsewhere [7]. This dose represents the median lethal dose or LD_50_, the dose required to kill 50% of the *T. molitor* larvae in a 14 d bioassay [20]. After 24 h, 23 control and 26 Cry3Aa-treated larvae were sacrificed and guts were collected in separate tubes of RNA*later*® (Ambion, Austin, TX USA). Gut tissues were processed to obtain total RNA (RNeasy®, Qiagen Inc., Valencia, CA USA) and the resulting samples were processed by 454 Life Sciences (a Roche Company, Branford, CT USA).

### Pyrosequencing

PolyA+ RNA was prepared from total RNA by using oligo(dT) magnetic beads (PureBiotech, LLC, Middlesex, NJ USA) and quantified with fluorometry. Single-stranded cDNA libraries were prepared as described elsewhere [52]. Briefly, 200 ng of mRNA were heat fragmented in a magnesium buffer and purified on a Sephadex G50 spin column (Roche Applied Science, Indianapolis, IN USA). First-strand cDNA was prepared from 400 pmol TNNT(N)6 oligo (Integrated DNA Technologies, Coralville, IA USA) using SuperScript® II reverse transcriptase (Invitrogen, Carlsbad, CA USA). Sodium hydroxide was used to melt cDNA/mRNA hybrids, and single-stranded cDNA was purified using the AMPure® purification kit (Agencourt Bioscience, Beverly, MA USA). Double-stranded adapters [52] were ligated to the single-stranded cDNA using T4 DNA Ligase (New England BioLabs, Ipswich, MA USA). Adapter-ligated cDNA libraries were isolated on Dynabeads® MyOne™ Streptavidin C1 (Invitrogen), and the single-stranded libraries were released by incubating in a 25 mM sodium hydroxide solution and purified using the AMPure® purification kit.

Sequencing was performed on the Genome Sequencer™ FLX pyrosequencing system according to the manufacturer’s instructions. Adaptor sequences were removed from raw sequences prior to data analysis.

### Sequence Assembly and Data Analysis

Sequence reads (258,377) were either separated by treatment type and independently assembled, or pooled and assembled, as described below.

For the first assembly, sequence reads were grouped into two datasets and assembled separately into contigs of 1,318 control and 1,140 Cry3Aa-treated (total nucleotides = 1,026,138 and 898,431; N50 contig size = 764 and 788; Q40+Bases = 88.5 and 88.23%, respectively), using the 454 De Novo Assembler (v1.1.03) with default parameters. Information regarding the number of sequence reads was integrated and retained within the contig files. Assembly 1 contigs were functionally annotated with Gene Ontology (GO) terms using Blast2GO [24]. Enrichment analysis was performed within the currently available platform in the program. In addition, an adaptation of the Fisher’s Exact test for functional enrichment analysis was developed to accept the read-length and number-of-reads values associated with each contig in the FASTA file from each assembly group (control and Cry3Aa-treated). In this analysis, for each functional category, the total number of reads present in contigs annotated to that function was normalized by the sum of contig lengths and was used to construct a contingency table of “function annotation/function not annotated” values for each dataset (data not shown). The Fisher’s Exact Test was applied to these data, and significant functional groups were obtained at a Benjamini and Hochberg [53] corrected FDR cut-off of 0.05, selecting a fold change >1, containing at least 2 contigs, and GO terms lacking significant child terms. To evaluate the most significant differences, GO terms were further restricted by scores >100.

A second assembly of pooled data (sequence reads from both the control and Cry3Aa-treated samples) was generated using SeqMan NGen® (DNASTAR, Inc., Madison, WI USA). Treated and control data were individually tagged to allow for downstream evaluation of log ratio values related to induced and repressed genes. Assembly parameters were optimized over time to obtain a final configuration and assembly (limiting false joins) with basic parameters of match size = 15, match percentage = 97%, match spacing = 25, maximum coverage = 10,000, and minimum coverage = 2. Assemblies were manually evaluated and edited using SeqMan (DNASTAR). Approximately 258,000 sequence reads (total number of reads obtained from the treated and untreated samples) were assembled into 25,201 EST contigs (uniESTs). Custom scripts were utilized to parse/determine the number and origin (control vs. Cry3Aa-treated) of each read used in generating the uniESTs.

### Microarray Design

A custom *T. molitor* microarray was developed using the uniESTs generated from the second assembly, with eArray software used for probe design (Agilent Technologies, Inc., Santa Clara, CA USA). Of the 25,201 uniESTs, 23,671 oligos were selected as unambiguous (with the program selecting specific oligos representative of each contig) and were arrayed in duplicate or triplicate on a custom array chip (4×44K, Agilent Technologies, Inc.), incorporating standards supplied by eArray. Sequences of oligos used in the microarray are presented in Table S6.

### Microarray Analysis

Newly molted *T. molitor* larvae (approximately 1 mo old and mean mass of 5–6 mg) were selected from a standard laboratory colony (reared on 50% rolled oats, 2.5% brewer’s yeast, 47.5% flour) and were starved overnight. Larvae were placed on a diet of 85% stabilized wheat germ, 10% flour, and 5% brewer’s yeast with a 3% dilution of a concentrated solution of FD&C Blue #1 and 97% of a mixture of 85% stabilized wheat germ, 10% flour, 5% brewer’s yeast with or without 0.1% (w/w) Cry3Aa (pre-equilibrated at 28°C, 75% R.H. over saturated sodium chloride). Larvae were monitored for blue guts every 30 min, and those with blue guts (containing either protoxin or not) were selected and reared further on diet containing protoxin or control diet, respectively, for 6, 12, or 24 h. Each corresponding time interval had a separate control and treatment. For each treatment, midguts from four larvae were dissected under and into RNA*later*® (Qiagen, Valencia, CA USA). A biological replicate was performed with larvae from a different oviposition tray.

Prior to RNA extraction, excess RNA*later*® was removed and guts were ground with a plastic pestle in a 1.5 ml microcentrifuge tube containing liquid nitrogen. Total RNA was isolated using the Absolutely RNA Kit, which includes on-column DNase treatment as described in the manufacturer’s protocol (Agilent Technologies, La Jolla, CA USA). Gut-specific mRNA was reverse transcribed from total RNA using an oligo-dT-T7 primer and amplified in the presence of Cy3 or Cy5-labelled dNTPs into complementary RNA (cRNA) using T7 RNA polymerase (Quick Amp Labeling Kit, Agilent), with dye swapping among the biological replicates. Hybridization of the *T. molitor* microarray was performed for 18 h at 45°C with approximately 825 ng of the modified cRNA (Gene Expression Hybridization Kit, Agilent). Microarrays were scanned at 532 and 635 nm using a GenePix 4000B scanner (Molecular Devices, Sunnyvale, CA USA) at the Gene Expression Facility at Kansas State University. Images were analyzed by Genepix Pro 6.1 software.

### Bioinformatic Analyses

For microarrays, relative intensity values from hybridizations (raw data) were imported into GeneSifter™ (Perkin Elmer, Seattle, WA USA) for statistical analysis. Data was normalized by relative intensity of the means and was log-transformed. Annotations associated with each oligo sequence in the statistically significant pool were verified by tBLASTx against NCBI’s non-redundant (nr) database. Pairwise comparisons were made between the control and each intoxication time-point dataset, and significance was determined by the Student’s t-test (p<0.05) and corrected by the Benjamini and Hochberg False Discovery Rate [53]. A comparison of control to all intoxication periods was by ANOVA (p<0.05), also correcting for FDR by the process of Benjamini and Hochberg [53]. Principle Component Analysis [54] was performed within GeneSifter™.

Multiple protein alignments and phylogenetic trees were from CLC Bio (Aarhus, Denmark). Signal peptide predictions were with Signal IP 4.0 [55].

### Quantitative PCR

Quantitative PCR (qPCR) was performed with two independent biological replicates of total RNA from *T. molitor* larval guts that were pooled from 10 larvae after 24 h on 0.1% Cry3Aa-treated diet (as described for the microarray analysis). qPCR was performed with 300 nM primer (Table S7) and 1 ng total RNA from either control or Cry3Aa-treated larvae and 2–3 technical replicates for each biological replicate, using Brilliant II SYBR Green qRT-PCR Master Mix Kit 1-Step (Agilent) in a Mx3000P qPCR thermocycler (Agilent). The thermal profile was: 30 min at 50°C; 10 min at 95°; 40 cycles of 30 sec at 95°, 60 sec at 55° and 30 sec at 72°; 60 sec at 95°; 30 sec at 55°; and 95° at 0.2°/sec to obtain dissociation curves. Relative quantity of transcripts expression in the Cry3-treated sample compared to control was normalized to ribosomal protein L24 (which had nearly identical Ct values for control and Cry3-treated samples that were within the range of Ct values observed for the genes of interest; data not shown).

## Supporting Information

Figure S1 **Alignment of predicted** ***Tribolium castaneum*** **sequences containing chitin-binding domain 3 (CBD3) from genes that are in tandem on chromosome 7 with predicted protein sequences of contigs from** ***Tenebrio molitor*** **.** Boxed regions contain sequences corresponding to microarray oligos from T. molitor contig sequences; yellow shaded regions correspond to the DNA that was amplified in qPCR. Signal peptides are predicted at the beginning of the CBD3 region, between G and H residues.
(TIF)

Figure S2 **Phylogenetic relationship of predicted proteins containing chitin-binding domain 3 from** ***Tribolium castaneum*** **and** ***Tenebrio molitor*** **.**
(TIF)

Table S1 **Enrichment analysis of Gene Ontology (GO) functions enriched in Cry3Aa-treated (Bt)** ***Tenebrio molitor*** **larvae compared to control (Control) larvae, as determined by Blast2GO analysis (Conesa et al., 2006).** Categories: BP, Biological Process; CC, Cellular Component; MF, Molecular Function. Total number of GO functions in the groups: Cry3Aa-treated, 16,395; Control, 22,696.
(DOCX)

Table S2 **Enrichment analysis of Gene Ontology (GO) functions repressed in Cry3Aa-treated (Bt)** ***Tenebrio molitor*** **larvae compared to control (Control) larvae, as determined by Blast2GO analysis (Conesa et al., 2006).** Categories: BP, Biological Process; CC, Cellular Component; MF, Molecular Function. Total number of GO functions in the groups: Cry3Aa-treated, 16,395; Control, 22,696.
(DOCX)

Table S3 **Pairwise analysis of significant (p<0.05) differences in gene expression in the gut of** ***Tenebrio molitor*** **larvae fed 0.1% Cry3Aa for 6 h compared to control larvae, as determined by microarray analysis.** Contig sequences are in Table S6.
(DOCX)

Table S4 **Pairwise analysis of significant (p<0.05) differences in gene expression in the gut of** ***Tenebrio molitor*** **larvae fed 0.1% Cry3Aa for 12 h compared to control larvae, as determined by microarray analysis.** Contig sequences are in Table S6.
(DOCX)

Table S5 **Pairwise analysis of significant (p<0.05) differences in gene expression in the gut of** ***Tenebrio molitor*** **larvae fed 0.1% Cry3Aa for 24 h compared to control larvae, as determined by microarray analysis and RNA-Seq.** Oligo sequences are in Table S6.
(DOCX)

Table S6 **Oligo sequences used in the microarray analysis, obtained from the second assembly of reads from high throughput sequencing of the** ***Tenebrio molitor*** **larval gut.**
(DOCX)

Table S7 **Primers used in qPCR to compare the expression of two CBD3 transcripts in Cry3Aa-intoxicated** ***Tenebrio molitor*** **larvae compared to control.**
(DOCX)

## References

[1] Schnepf E, Crickmore N, Van Rie J, Lereclus D, Baum J (1998). *Bacillus thuringiensis* and its pesticidal crystal proteins.. Microbiol Mol Biol Rev.

[2] Shelton AM, Zhao JZ, Roush RT (2002). Economic, ecological, food safety, and social consequences of the deployment of Bt transgenic plants.. Ann Rev Entomol.

[3] Crickmore N, Zeigler DR, Schnepf E, Van Rie J, Lereclus D (2011). *Bacillus thuringiensis* toxin nomenclature.

[4] van Frankenhuyzen K (2009). Insecticidal activity of *Bacillus thuringiensis* crystal proteins.. J Invertebr Pathol.

[5] Bravo A, Gill SS, Soberón M (2007). Mode of action of *Bacillus thuringiensis* Cry and Cyt toxins and their potential for insect control.. Toxicon.

[6] Zhang X, Candas M, Griko NB, Taussig R, Bulla LA (2006). A mechanism of cell death involving an adenylyl cyclase/PKA signaling pathway is induced by the Cry1Ab toxin of *Bacillus thuringiensis*.. Proc Natl Acad Sci U S A.

[7] Fabrick J, Oppert C, Lorenzen MD, Morris K, Oppert B (2009). A novel *Tenebrio molitor* cadherin is a functional receptor for *Bacillus thuringiensis* Cry3Aa toxin.. J Biol Chem.

[8] Huffman DL, Abrami L, Sasik R, Corbeil J, van der Goot FG (2004). Mitogen-activated protein kinase pathways defend against bacterial pore-forming toxins.. Proc Natl Acad Sci U S A.

[9] Bischof LJ, Kao CY, Los FC, Gonzalez MR, Shen Z (2008). Activation of the unfolded protein response is required for defenses against bacterial pore-forming toxin *in vivo*.. PLoS Path.

[10] Chen CS, Bellier A, Kao CY, Yang YL, Chen HD (2010). WWP-1 is a novel modulator of the DAF-2 insulin-like signaling network involved in pore-forming toxin cellular defenses in *Caenorhabditis elegans*.. PLoS ONE.

[11] Bellier A, Chen CS, Kao CY, Cinar HN, Aroian RV (2009). Hypoxia and the hypoxic response pathway protect against pore-forming toxins in C. elegans.. PLoS Pathog.

[12] Kao CY, Los FC, Huffman DL, Wachi S, Kloft N (2011). Global functional analyses of cellular responses to pore-forming toxins.. PLoS Pathog.

[13] Meunier L, Préfontaine G, Van Munster M, Brousseau R, Masson L (2006). Transcriptional response of *Choristoneura fumiferana* to sublethal exposure of Cry1Ab protoxin from *Bacillus thuringiensis*.. Insect Mol Biol.

[14] van Munster M, Prefontaine G, Meunier L, Elias M, Mazza A (2007). Altered gene expression in *Choristoneura fumiferana* and *Manduca sexta* in response to sublethal intoxication by *Bacillus thuringiensis* Cry1Ab toxin.. Insect Mol Biol.

[15] Khajuria C, Zhu YC, Chen MS, Buschman LL, Higgins RA (2009). Expressed sequence tags from larval gut of the European corn borer (*Ostrinia nubilalis*): Exploring candidate genes potentially involved in *Bacillus thuringiensis* toxicity and resistance.. BMC Genomics.

[16] Hernández-Martínez P, Navarro-Cerrillo G, Caccia S, de Maagd RA, Moar WJ (2010). Constitutive activation of the midgut response to *Bacillus thuringiensis* in Bt-resistant *Spodoptera exigua*.. PLoS ONE.

[17] Paris M, Melodelima C, Coissac E, Tetreau G, Reynaud S (2011). Transcription profiling of resistance to Bti toxins in the mosquito *Aedes aegypti* using next-generation sequencing..

[18] Richards S, Gibbs RA, Weinstock GM, Brown SJ, Denell R (2008). The genome of the model beetle and pest *Tribolium castaneum*.. Nature.

[19] Morris K, Lorenzen MD, Hiromasa Y, Tomich JM, Oppert C (2009). The *Tribolium castaneum* larval gut transcriptome and proteome: A resource for the study of the coleopteran gut.. J Proteome Res.

[20] Oppert B, Morgan TD, Kramer KJ (2011). Efficacy of *Bacillus thuringiensis* Cry3Aa protoxin and protease inhibitors against coleopteran storage pests.. Pest Manage Sci.

[21] Carroll J, Li J, Ellar DJ (1989). Proteolytic processing of a coleopteran-specific delta-endotoxin produced by *Bacillus thuringiensis* var. *tenebrionis*.. Biochem J.

[22] Wu S-J, Dean DH (1996). Functional significance of loops in the receptor binding domain of *Bacillus thuringiensis* CryIIIA δ-endotoxin.. J Mol Biol.

[23] Prabhakar S, Chen MS, Elpidina EN, Vinokurov KS, Smith CM (2007). Sequence analysis and molecular characterization of larval midgut cDNA transcripts encoding peptidases from the yellow mealworm, *Tenebrio molitor* L. Insect Mol Biol.

[24] Conesa A, Gotz S, Garcia-Gomez JM, Terol J, Talon M (2005). Blast2GO: a universal tool for annotation, visualization and analysis in functional genomics research.. Bioinformatics.

[25] Ashburner M, Ball CA, Blake JA, Botstein D, Butler H (2000). Gene ontology: tool for the unification of biology. The Gene Ontology Consortium.. Nat Genet.

[26] Morgan NS, Heintzelman MB, Mooseker MS (1995). Characterization of myosin-IA and myosin-IB, two unconventional myosins associated with the *Drosophila* brush border cytoskeleton.. Dev Biol.

[27] Fischer HM, Wheat CW, Heckel DG, Vogel H (2008). Evolutionary origins of a novel host plant detoxification gene in butterflies.. Mol Biol Evol.

[28] Chintapalli VR, Wang J, Dow JA (2007). Using FlyAtlas to identify better *Drosophila melanogaster* models of human disease.. Nat Genet.

[29] Cristofoletti PT, Ribeiro AF, Terra WR (2005). The cathepsin L-like proteinases from the midgut of *Tenebrio molitor* larvae: sequence, properties, immunocytochemical localization and function.. Insect Biochem Mol Biol.

[30] Vinokurov KS, Elpidina EN, Oppert B, Prabhakar S, Zhuzhikov DP (2006). Diversity of digestive proteinases in *Tenebrio molitor* (Coleoptera: Tenebrionidae) larvae.. Comp Biochem Physiol B Biochem Mol Biol.

[31] Zhao M, Soderhall I, Park JW, Ma YG, Osaki T (2005). A novel 43-kDa protein as a negative regulatory component of phenoloxidase-induced melanin synthesis.. J Biol Chem.

[32] Levashina EA (2004). Immune responses in *Anopheles gambiae*.. Insect Biochem Mol Biol.

[33] Nguyen G, Delarue F, Burckle C, Bouzhir L, Giller T (2002). Pivotal role of the renin/prorenin receptor in angiotensin II production and cellular responses to renin.. J Clin Invest.

[34] Park SG, Schimmel P, Kim S (2008). Aminoacyl tRNA synthetases and their connections to disease.. Proc Natl Acad Sci U S A.

[35] Altincicek B, Knorr E, Vilcinskas A (2008). Beetle immunity: Identification of immune-inducible genes from the model insect *Tribolium castaneum*.. Dev Comp Immunol.

[36] Zou Z, Evans JD, Lu Z, Zhao P, Williams M (2007). Comparative genomic analysis of the *Tribolium* immune system.. Genome Biol.

[37] Eriksson TL, Whitley P, Johansson E, van Hage-Hamsten M, Gafvelin G (1999). Identification and characterisation of two allergens from the dust mite *Acarus siro*, homologous with fatty acid-binding proteins.. Int Arch Allergy Immunol.

[38] Annaert WG, Becker B, Kistner U, Reth M, Jahn R (1997). Export of cellubrevin from the endoplasmic reticulum is controlled by BAP31.. J Cell Biol.

[39] Nguyen M, Breckenridge DG, Ducret A, Shore GC (2000). Caspase-resistant BAP31 inhibits fas-mediated apoptotic membrane fragmentation and release of cytochrome c from mitochondria.. Mol Cell Biol.

[40] Buchon N, Broderick NA, Poidevin M, Pradervand S, Lemaitre B (2009). *Drosophila* intestinal response to bacterial infection: activation of host defense and stem cell proliferation.. Cell Host Microbe.

[41] Pigott CR, Ellar DJ (2007). Role of receptors in *Bacillus thuringiensis* crystal toxin activity.. Microbiol Mol Biol Rev.

[42] Sayed A, Wiechman B, Struewing I, Smith M, French W (2010). Isolation of transcripts from *Diabrotica virgifera virgifera* LeConte responsive to the *Bacillus thuringiensis* toxin Cry3Bb1.. Insect Mol Biol.

[43] Lee KH, Hong SY, Oh JE, Kwon M, Yoon JH (1998). Identification and characterization of the antimicrobial peptide corresponding to C-terminal beta-sheet domain of tenecin 1, an antibacterial protein of larvae of *Tenebrio molitor*.. Biochem J 334.

[44] Rodriguez Mdel C, Martinez-Barnetche J, Alvarado-Delgado A, Batista C, Argotte-Ramos RS (2007). The surface protein Pvs25 of *Plasmodium vivax* ookinetes interacts with calreticulin on the midgut apical surface of the malaria vector *Anopheles albimanus*.. Mol Biochem Parasitol.

[45] Weers PM, Ryan RO (2006). Apolipophorin III: role model apolipoprotein.. Insect Biochem Mol Biol.

[46] Jurat-Fuentes JL, Adang MJ (2004). Characterization of a Cry1Ac-receptor alkaline phosphatase in susceptible and resistant *Heliothis virescens* larvae.. Eur J Biochem.

[47] Tiewsiri K, Wang P (2011). Differential alteration of two aminopeptidases N associated with resistance to *Bacillus thuringiensis* toxin Cry1Ac in cabbage looper.. Proc Natl Acad Sci U S A.

[48] Ochoa-Campuzano C, Real MD, Martínez-Ramírez AC, Bravo A, Rausell C (2007). An ADAM metalloprotease is a Cry3Aa *Bacillus thuringiensis* toxin receptor.. Biochem Biophys Res Commun.

[49] Heckel DG, Gahan LJ, Baxter SW, Zhao JZ, Shelton AM (2007). The diversity of Bt resistance genes in species of Lepidoptera.. J Invertebr Pathol.

[50] Johannessen BR, Skov LK, Kastrup JS, Kristensen O, Bolwig C (2005). Structure of the house dust mite allergen Der f 2: implications for function and molecular basis of IgE cross-reactivity.. FEBS Lett.

[51] Cancino-Rodezno A, Alexander C, Villasenor R, Pacheco S, Porta H (2010). The mitogen-activated protein kinase p38 is involved in insect defense against Cry toxins from *Bacillus thuringiensis*.. Insect Biochem Mol Biol.

[52] Sugarbaker DJ, Richards WG, Gordon GJ, Dong L, De Rienzo A (2008). Transcriptome sequencing of malignant pleural mesothelioma tumors.. Proc Natl Acad Sci U S A.

[53] Benjamini Y, Hochberg Y (1995). Controlling the False Discovery Rate - a practical and powerful approach to multiple testing.. J Roy Stat Soc B Met.

[54] Pearson K (1901). On lines and planes of closest fit to systems of points in space.. Philos Mag.

[55] Petersen TN, Brunak S, von Heijne G, Nielsen H (2011). SignalP 4.0: discriminating signal peptides from transmembrane regions.. Nat Methods.

