# Supplementary figures and images for: Transcriptome Profiling of the Intoxication Response of Tenebrio molitor Larvae to Bacillus thuringiensis Cry3Aa Protoxin
Source: PLoS One. 2012 Apr 25;7(4):e34624. doi: 10.1371/journal.pone.0034624 (PMC3338813; doi:10.1371/journal.pone.0034624)

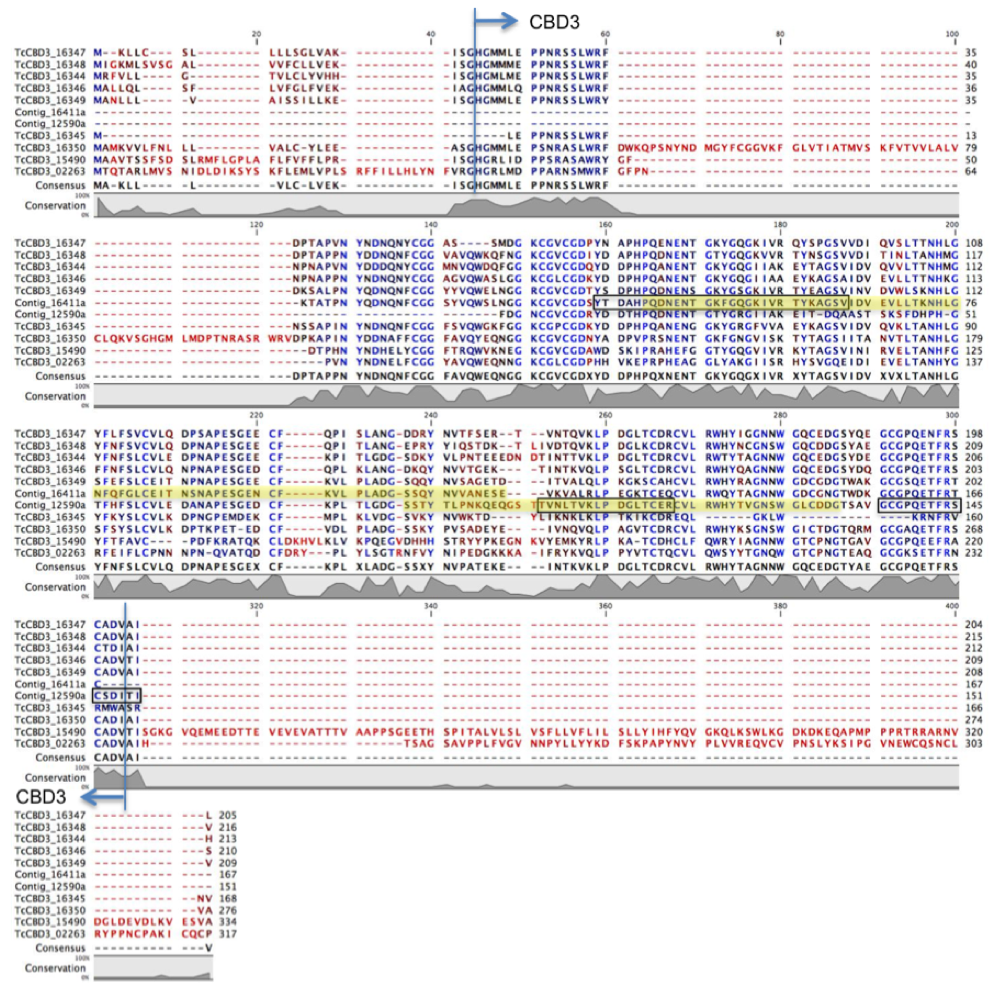

Supplement: Figure S1 — Alignment of predicted Tribolium castaneum sequences containing chitin-binding domain 3 (CBD3) from genes that are in tandem on chromosome 7 with predicted protein sequences of contigs from Tenebrio molitor . Boxed regions contain sequences corresponding to microarray oligos from T. molitor contig sequences; yellow shaded regions correspond to the DNA that was amplified in qPCR. Signal peptides are predicted at the beginning of the CBD3 region, between G and H residues. (TIF) [file pone.0034624.s001.tif]

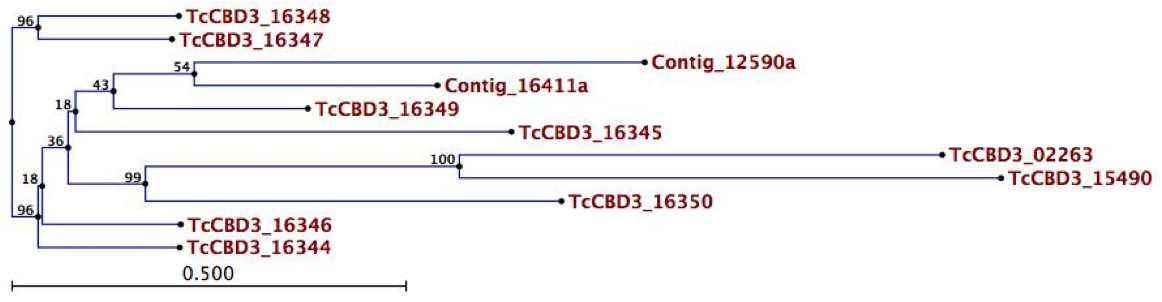

Supplement: Figure S2 — Phylogenetic relationship of predicted proteins containing chitin-binding domain 3 from Tribolium castaneum and Tenebrio molitor . (TIF) [file pone.0034624.s002.tif]
