# Supplementary material for: Transcriptome Profiling of the Intoxication Response of Tenebrio molitor Larvae to Bacillus thuringiensis Cry3Aa Protoxin
Source: PLoS One. 2012 Apr 25;7(4):e34624. doi: 10.1371/journal.pone.0034624 (PMC3338813; doi:10.1371/journal.pone.0034624)
Supplement: Table S1 — Enrichment analysis of Gene Ontology (GO) functions enriched in Cry3Aa-treated (Bt) Tenebrio molitor larvae compared to control (Control) larvae, as determined by Blast2GO analysis (Conesa et al., 2006). Categories: BP, Biological Process; CC, Cellular Component; MF, Molecular Function. Total number of GO functions in the groups: Cry3Aa-treated, 16,395; Control, 22,696. (DOCX) [file pone.0034624.s003.docx]

## Table S1.

| **GO ID_Category** | **GO Description** | **Control^a^** | **Bt^a^** | **#Control Contigs** | **#Bt Contigs** |
| --- | --- | --- | --- | --- | --- |
| GO:0015992_BP | proton transport | 799 | 3989 | 13 | 22 |
| GO:0045277_CC | respiratory chain complex IV | 0 | 2233 | 0 | 5 |
| GO:0023046_BP | signalling | 1041 | 2117 | 38 | 54 |
| GO:0042254_BP | ribosome biogenesis | 423 | 1411 | 10 | 38 |
| GO:0006814_BP | sodium ion transport | 60 | 1248 | 2 | 4 |
| GO:0006744_BP | ubiquinone biosynthetic process | 0 | 1151 | 0 | 3 |
| GO:0005667_CC | transcription factor complex | 23 | 533 | 1 | 21 |
| GO:0045298_CC | tubulin complex | 14 | 255 | 1 | 7 |
| GO:0006448_BP | regulation of translational elongation | 12 | 247 | 1 | 5 |
| GO:0006446_BP | regulation of translational initiation | 0 | 222 | 0 | 6 |
| GO:0043565_MF | sequence-specific DNA binding | 85 | 217 | 5 | 11 |
| GO:0042803_MF | protein homodimerization activity | 102 | 214 | 4 | 6 |
| GO:0009069_BP | serine family amino acid metabolic process | 99 | 212 | 5 | 12 |
| GO:0007005_BP | mitochondrion organization | 66 | 206 | 3 | 6 |
| GO:0016051_BP | carbohydrate biosynthetic process | 86 | 205 | 5 | 6 |
| GO:0006357_BP | regulation of transcription from RNA polymerase II promoter | 47 | 205 | 3 | 7 |
| GO:0007318_BP | pole plasm protein localization | 54 | 200 | 2 | 5 |
| GO:0002119_BP | nematode larval development | 44 | 196 | 3 | 5 |
| GO:0005874_CC | microtubule | 79 | 193 | 4 | 5 |
| GO:0005982_BP | starch metabolic process | 0 | 187 | 0 | 3 |
| GO:0005985_BP | sucrose metabolic process | 0 | 187 | 0 | 3 |
| GO:0051258_BP | protein polymerization | 69 | 177 | 3 | 5 |
| GO:0016998_BP | cell wall macromolecule catabolic process | 0 | 166 | 0 | 3 |
| GO:0003702_MF | RNA polymerase II transcription factor activity | 32 | 165 | 2 | 4 |
| GO:0045169_CC | fusome | 35 | 163 | 2 | 5 |
| GO:0045941_BP | positive regulation of transcription, DNA-dependent | 23 | 158 | 1 | 7 |
| GO:0008277_BP | regulation of G-protein coupled receptor protein signalling pathway | 58 | 146 | 1 | 3 |
| GO:0005865_CC | striated muscle thin filament | 70 | 143 | 2 | 3 |
| GO:0006839_BP | mitochondrial transport | 68 | 143 | 3 | 6 |
| GO:0045859_BP | regulation of protein kinase activity | 26 | 137 | 2 | 4 |
| GO:0003712_MF | transcription cofactor activity | 67 | 135 | 4 | 5 |
| GO:0031145_BP | proteasomal ubiquitin-dependent protein catabolic process | 63 | 134 | 1 | 4 |
| GO:0016057_BP | regulation of membrane potential in photoreceptor cell | 17 | 126 | 1 | 3 |
| GO:0035003_CC | subapical complex | 17 | 126 | 1 | 3 |
| GO:0007088_BP | regulation of mitosis | 29 | 124 | 1 | 3 |
| GO:0006568_BP | tryptophan metabolic process | 59 | 119 | 1 | 4 |
| GO:0007317_BP | regulation of pole plasm oskar mRNA localization | 53 | 107 | 2 | 4 |
| GO:0006470_BP | protein dephosphorylation | 20 | 105 | 1 | 3 |
| GO:0035062_CC | omega speckle | 23 | 105 | 1 | 3 |

^a^Scores from an enrichment analysis of total GO functions in each dataset, normalized by the length and the number of reads associated with each contig, using the Fisher Exact Test, p<0.05. Data was filtered by scores>100, fold-change>1, and number of contigs >2 in the Cry3Aa-treated dataset, and selecting for parent terms without significant child terms.
