# Supplementary material for: Transcriptome Profiling of the Intoxication Response of Tenebrio molitor Larvae to Bacillus thuringiensis Cry3Aa Protoxin
Source: PLoS One. 2012 Apr 25;7(4):e34624. doi: 10.1371/journal.pone.0034624 (PMC3338813; doi:10.1371/journal.pone.0034624)
Supplement: Table S2 — Enrichment analysis of Gene Ontology (GO) functions repressed in Cry3Aa-treated (Bt) Tenebrio molitor larvae compared to control (Control) larvae, as determined by Blast2GO analysis (Conesa et al., 2006). Categories: BP, Biological Process; CC, Cellular Component; MF, Molecular Function. Total number of GO functions in the groups: Cry3Aa-treated, 16,395; Control, 22,696. (DOCX) [file pone.0034624.s004.docx]

## Table S2.

| **GO ID_Category** | **GO Description** | **Control^a^** | **Cry3Aa-treated^a^** | **#Control Contigs** | **#Bt Contigs** |
| --- | --- | --- | --- | --- | --- |
| GO:0008237_MF | metallopeptidase activity | 286 | 10 | 5 | 1 |
| GO:0006096_BP | glycolysis | 271 | 40 | 9 | 2 |
| GO:0007298_BP | border follicle cell migration | 231 | 48 | 6 | 2 |
| GO:0005777_CC | peroxisome | 215 | 42 | 6 | 2 |
| GO:0032027_MF | myosin light chain binding | 192 | 25 | 4 | 1 |
| GO:0032956_BP | regulation of actin cytoskeleton organization | 189 | 51 | 3 | 2 |
| GO:0016209_MF | antioxidant activity | 179 | 43 | 7 | 2 |
| GO:0034331_BP | cell junction maintenance | 170 | 25 | 3 | 1 |
| GO:0006281_BP | DNA repair | 168 | 22 | 3 | 1 |
| GO:0009119_BP | ribonucleoside metabolic process | 159 | 0 | 3 | 0 |
| GO:0043648_BP | dicarboxylic acid metabolic process | 149 | 11 | 4 | 1 |
| GO:0034656_BP | nucleobase, nucleoside and nucleotide catabolic process | 146 | 37 | 3 | 2 |
| GO:0045597_BP | positive regulation of cell differentiation | 141 | 0 | 3 | 0 |
| GO:0051240_BP | positive regulation of multicellular organismal process | 125 | 0 | 3 | 0 |
| GO:0000287_MF | magnesium ion binding | 113 | 24 | 6 | 2 |
| GO:0008415_MF | transferase activity, transferring acyl groups | 112 | 27 | 6 | 2 |
| GO:0051098_BP | regulation of binding | 103 | 26 | 3 | 2 |
| GO:0015928_MF | fucosidase activity | 100 | 23 | 3 | 1 |

^a^Scores from an enrichment analysis of total GO functions in each dataset, normalized by the length and the number of reads associated with each contig, using the Fisher Exact Test, p<0.05. Data was filtered by scores>100, fold-change>1, and number of contigs >2 in the Control dataset, and selecting for parent terms without significant child terms.
