# Supplementary material for: Transcriptome Profiling of the Intoxication Response of Tenebrio molitor Larvae to Bacillus thuringiensis Cry3Aa Protoxin
Source: PLoS One. 2012 Apr 25;7(4):e34624. doi: 10.1371/journal.pone.0034624 (PMC3338813; doi:10.1371/journal.pone.0034624)
Supplement: Table S3 — Pairwise analysis of significant (p<0.05) differences in gene expression in the gut of Tenebrio molitor larvae fed 0.1% Cry3Aa for 6 h compared to control larvae, as determined by microarray analysis. Contig sequences are in Table S6. (DOCX) [file pone.0034624.s005.docx]

## Table S3.

| **Contig #** | **Best Hit^a^** | **Predicted Function^a^** | **Fold Difference^b^** |
| --- | --- | --- | --- |
| **18860** | AY327800^c^ CG10477 | cockroach allergen-like protein | 8.37 |
| **16751** | TC013662 CG15918 | chitin deacetylase 6 | 2.02 |
| **16243** | TC004032 CG14949 | na | 2.02 |
| **18326** | AY337517^c^ Cp1, TC009365 | cathepsin L-like protein | 1.94 |
| **7267** | TC007858 | Protein of unknown function (DUF3421) | 1.71 |
| **14493** | TC010829 betaTub56D | beta tubulin | 1.57 |
| **19654** | TC010829 betaTub97EF | beta tubulin | 1.53 |
| **8689** | XM_965528 | vacuolar ATP synthase | 1.50 |
| **262** | TC013519 | lupus 1a ribonucleoprotein | 1.48 |
| **21756** | TC012005 CG12099 | RING-finger protein 10 | 1.30 |
| **17333** | XM_961998 | juvenile hormone-inducible protein | 1.23 |
| **21958** | TC013754 | renin receptor-like protein | -1.13 |
| **6467** | TC013555 | transcription initiation factor | -1.24 |
| **7075** | TC008409 | translation machinery associated protein mct-1 | -1.27 |
| **11225** | TC012531 | aminoacylase | -1.35 |
| **16635** | XM_002523507 | serine-threonine protein kinase | -1.54 |
| **15484** | TC004597 | synaptic vesicle protein | -1.72 |
| **2187** | XM_967458 GA13327 | proteasome | -1.90 |
| **4230** | AB205184^c^ Sr-CI | melanin-inhibiting protein | -1.98 |

^a^BLAST hits are from TBLASTX of contigs with NCBI nr, filtering <e^-0.05^, including the *D. melanogaster* or *T. castaneum* ortholog when available, with the predicted function based on sequence homology when available; na-no associated sequence and/or function.

^b^Fold difference is the relative expression in Cry3Aa-intoxicated larvae compared to control.

^c^*Tenebrio molitor*
