# Supplementary material for: Transcriptome Profiling of the Intoxication Response of Tenebrio molitor Larvae to Bacillus thuringiensis Cry3Aa Protoxin
Source: PLoS One. 2012 Apr 25;7(4):e34624. doi: 10.1371/journal.pone.0034624 (PMC3338813; doi:10.1371/journal.pone.0034624)
Supplement: Table S4 — Pairwise analysis of significant (p<0.05) differences in gene expression in the gut of Tenebrio molitor larvae fed 0.1% Cry3Aa for 12 h compared to control larvae, as determined by microarray analysis. Contig sequences are in Table S6. (DOCX) [file pone.0034624.s006.docx]

## Table S4.

| **Contig #** | **Best Hit^a^** | **Predicted Function^a^** | **Fold Difference^b^** |
| --- | --- | --- | --- |
| **12590** | TC016344/6 CG4367 | chitin-binding domain 3 | 9.88 |
| **20445** | TC007958 | na | 2.74 |
| **19676** | DQ356028^c^, TC016372 | serine protease homolog | 2.15 |
| **13451** | XM_964186 CG15117 | glycosyl hydrolase | 2.11 |
| **7267** | TC007858 | Protein of unknown function (DUF3421) | 1.47 |
| **20870** | TC013187 | Rho family small GTP binding protein cdc42 | 1.46 |
| **17333** | XM_961998 | juvenile hormone-inducible protein | 1.43 |
| **14493** | TC010829 betaTub56D | beta tubulin | 1.40 |
| **19654** | TC010829 betaTub97EF | beta tubulin | 1.38 |
| **262** | TC013519 | lupus la ribonucleoprotein | 1.37 |
| **9828** | XM_969848 CG17347 | dynactin | 1.29 |
| **3209** | TC016342 | tryptophanyl-tRNA synthetase/ligase (Interferon-induced protein 53) | 1.29 |
| **10177** | TC009539 CG11738 | lethal (1) G0004; RNA-binding | 1.27 |
| **3936** | TC012876 Ntf-2 | nuclear transport factor-2 | 1.27 |
| **18373** | XM_966328 CG6843 | IP14452p, transcription initiation factor | 1.26 |
| **6626** | XM_317046^d^ | mitochondrial ribosomal protein L24 | 1.24 |
| **10290** | TC006245 | suppressor of Ty 4 homolog 1; transcription elongation factor SPT4 | 1.21 |
| **21958** | TC013754 | renin receptor-like protein | -1.16 |
| **17985** | TC006158 | na | -1.22 |
| **12482** | TC008729 | thiolase; acetyl transferase | -1.34 |
| **13321** | TC010472 CG10753 | small nuclear ribonucleoprotein at 69D | -1.36 |
| **12201** | TC010448 | 6-phosphogluconate dehydrogenase | -1.41 |
| **11374** | TC011289 | GTPase subunit SAR (Secretion associated, Ras-related) | -1.50 |
| **6914** | XM_961851 | inhibitor of NFkappaB kinase | -1.56 |
| **23242** | TC014564 | adenosylhomocysteinase | -1.61 |
| **19513** | TC004948 | peroxiredoxin | -1.82 |
| **4188** | TC015224 | serpin peptidase inhibitor 31 | -1.86 |
| **2107** | HP570794^e^ | coiled-coil domain containing 94 | -1.88 |
| **1448** | XM_963464 | phospholipid scramblase 1 | -2.10 |
| **4024** | XM_968699 | na | -2.12 |
| **7506** | TC014024 | TLD, TBC domain | -2.17 |
| **12173** | XM_962191 | esterase | -2.57 |
| **16145** | AY325895^c^ | chitinase | -2.66 |
| **13233** | XM_963136 | xanthine dehydrogenase | -2.88 |
| **15790** | AB021700^c^ | 86 kDa early-staged encapsulation inducing protein | -2.88 |
| **11938** | TC015577 | Pleiohomeotic (Pho) | -3.05 |
| **5757** | TC006775 | histone-binding protein RBBP4 (retinoblastoma-binding protein 4) | -3.19 |
| **3048** | TC004656 | lipase | -4.54 |

^a^BLAST hits are from TBLASTX of contigs with NCBI nr, filtering <e^-0.05^, including the *D. melanogaster* or *T. castaneum* ortholog when available, with the predicted function based on sequence homology when available; na-no associated sequence and/or function.

^b^Fold difference is the relative expression in Cry3Aa-intoxicated larvae compared to control.

^c^*Tenebrio molitor*

^d^*Anopheles gambiae*

^e^*Apis mellifera*
