# Supplementary material for: Transcriptome Profiling of the Intoxication Response of Tenebrio molitor Larvae to Bacillus thuringiensis Cry3Aa Protoxin
Source: PLoS One. 2012 Apr 25;7(4):e34624. doi: 10.1371/journal.pone.0034624 (PMC3338813; doi:10.1371/journal.pone.0034624)
Supplement: Table S5 — Pairwise analysis of significant (p<0.05) differences in gene expression in the gut of Tenebrio molitor larvae fed 0.1% Cry3Aa for 24 h compared to control larvae, as determined by microarray analysis and RNA-Seq. Oligo sequences are in Table S6. (DOCX) [file pone.0034624.s007.docx]

## Table S5.

| **Contig #** | **Best Hit^a^** | **Predicted Function^a^** | **Relative Fold Difference** | |
| --- | --- | --- | --- | --- |
|  |  |  | **Microarray^b^** | **RNA-Seq^c^** |
| **12590** | TC016344/6 CG4367 | chitin-binding domain 3 | 24.6 | 1.62 |
| **18860** | AY327800^d^ CG10477 | cockroach allergen-like protein | 7.42 | 2.92 |
| **12894** | TC011564 | thaumatin | 5.80 | 1.00 |
| **20445** | TC007958 | na | 4.58 | 1.39 |
| **16243** | TC004032 CG14949 | na | 3.96 | 2.00 |
| **20794** | TC016344 CG4367 | chitin-binding domain 3 | 3.86 | 1.00 |
| **20629** | AF312017^d^ CG9701 | β-glucosidase | 3.58 | 2.00 |
| **9375** | AY337517^d^ Cp1, TC009365 | cathepsin L | 2.82 | 1.09 |
| **16973** | DQ356032^d^ TC013672 | serine protease | 2.30 | 2.46 |
| **16** | TC000515 | thaumatin | 2.12 | 2.26 |
| **18492** | DQ356032^d^ TC013672 | serine protease | 2.04 | 2.00 |
| **4230** | AB205184^d^ Sr-CI | melanin-inhibiting protein | 1.82 | -1.13 |
| **16751** | TC013662 CG15918 | chitin deacetylase 6 | 1.68 | 1.39 |
| **19702** | TC005795 CG1244 | na | 1.62 | -1.32 |
| **19654** | TC010829 betaTub97EF | beta tubulin | 1.35 | 1.39 |
| **373** | XM_965079 | osteoadherin | 1.31 | 1.39 |
| **20501** | TC007958 | na | 1.28 | 1.00 |
| **866** | TC000030 Ard1 | α-N-acetyltransferase | -1.12 | 1.18 |
| **9624** | TC008958 CG8331 | receptor expression enhancing protein | -1.15 | 1.23 |
| **12948** | XM_970914 | na | -1.21 | 1.00 |
| **5818** | TC000069 Pros26 | proteasome | -1.23 | -1.52 |
| **2974** | XM_971400 | na | -1.30 | -1.23 |
| **3906** | TC003872 Fbp2 | alcohol dehydrogenase | -1.44 | -1.39 |
| **8122** | XM_001811882 GA12600 | B-cell receptor-associated protein 31-like | -1.51 | -1.48 |
| **7638** | TC009981 CG9471 | NADPH dehydrogenase | -1.69 | -1.39 |
| **21620** | TC001310 | allergen Aca s 13 | -2.40 | 2.00 |
| **3746** | TC012575 CG11529 | serine protease | -2.56 | -3.04 |
| **16411** | XM_963397 CG4367 | chitin-binding domain 3 | -5.68 | -1.49 |

^a^BLAST hits are from TBLASTX of contigs with NCBI nr, filtering <e^-0.05^, including the *D. melanogaster* or *T. castaneum* ortholog when available, with the predicted function based on sequence homology when available; na-no associated sequence and/or function.

^b^Microarray fold difference is the relative expression in Cry3Aa-intoxicated larvae compared to control.

^c^RNA-Seq fold difference is relative fold increase based on the number of reads from pyrosequencing of larvae exposed to Cry3Aa for 24 h compared to unexposed control larvae.

^d^*Tenebrio molitor*
