# Supplementary material for: Transcriptome Profiling of the Intoxication Response of Tenebrio molitor Larvae to Bacillus thuringiensis Cry3Aa Protoxin
Source: PLoS One. 2012 Apr 25;7(4):e34624. doi: 10.1371/journal.pone.0034624 (PMC3338813; doi:10.1371/journal.pone.0034624)
Supplement: Table S6 — Oligo sequences used in the microarray analysis, obtained from the second assembly of reads from high throughput sequencing of the Tenebrio molitor larval gut. (DOCX) [file pone.0034624.s008.docx]

**Table S6.**

| **Contig #** | **Best Hit^a^** | **Predicted Function^a^** | **Fold Difference^b^** | **RNA-Seq^c^** | **Oligo** |
| --- | --- | --- | --- | --- | --- |
| **16** | TC000515 | thaumatin | 2.12 | 2.26 | TTACAATATCCGGATATCTTTGGAGCCGATTAATGGACAAGGTGACGGTAGCGAGTACAG |
|  |  |  |  |  | GTTACAATATCCGGATATCTTTGGAGCCGATTAATGGACAAGGTGACGGTAGCGAGTACA |
| **262** | TC013519 | lupus 1a ribonucleoprotein | 1.48 |  | CTTTGGAGGTATTATTAACGTTCAAGAGACTTGCCAGTTTATCTGATGATCCAGAAGTGA |
|  |  |  |  |  | TTGCCAGTTTATCTGATGATCCAGAAGTGATAGCTTCTGCAGTGGAAAAAGCTGAAAATG |
| **262** | TC013519 | lupus la ribonucleoprotein | 1.37 |  | CTTTGGAGGTATTATTAACGTTCAAGAGACTTGCCAGTTTATCTGATGATCCAGAAGTGA |
|  |  |  |  |  | TTGCCAGTTTATCTGATGATCCAGAAGTGATAGCTTCTGCAGTGGAAAAAGCTGAAAATG |
| **373** | XM_965079 | osteoadherin | 1.31 | 1.39 | ATTCAGGATCTGACTTGGGACGAATTTGAAGGTCTGGCCAATCTCGAGGTATTGAATGTT |
|  |  |  |  |  | GCACAAATCGAATTTCGTCTTTTGATGCTGACAAAATTGCTACGAACTTCCTCAATCTAA |
| **1448** | XM_963464 | phospholipid scramblase 1 | -2.1 |  | TACTATGATTGATCAGTTGCTAGTCCACCAAAAAAGTCGAGTTGCTGGAAGCTCTTACCG |
|  |  |  |  |  | TTACTATGATTGATCAGTTGCTAGTCCACCAAAAAAGTCGAGTTGCTGGAAGCTCTTACC |
| **2107** | HP570794 | coiled-coil domain containing 94 | -1.88 |  | ATATACCGATTCTATATCAAATGCACCAGGTGCCTGCAGGAGATTTCGTTCAAGACAGAC |
|  |  |  |  |  | TATACCGATTCTATATCAAATGCACCAGGTGCCTGCAGGAGATTTCGTTCAAGACAGACC |
| **2187** | XM_967458 GA13327 | proteasome | -1.9 |  | AATGTTGGAGCTCCTCAACCAACTGGACGGCTTCGAAGCCACCAAAAACATCAAAGTTAT |
|  |  |  |  |  | CATCAAAGTTATCATGGCCACCAACAGAATTGACATACTGGATCCCGCCTTGTTAAGACC |
| **2974** | XM_971400 | na | -1.3 | -1.23 | CTATTTGCAGTTGATAAGTCGTTTGAAAGCGAGAGAAGAGGCGCGAGAAGATGAGTTGTA |
|  |  |  |  |  | TTGTCATCTACCAAAGCAGGCTATTTGCAGTTGATAAGTCGTTTGAAAGCGAGAGAAGAG |
| **3048** | TC004656 | lipase | -4.54 |  | ATCTTTGGAGATTACTATCTGGATACCAACGCCGACTCCCCATACGGACAAGGTTAGAAT |
|  |  |  |  |  | TCTTTGGAGATTACTATCTGGATACCAACGCCGACTCCCCATACGGACAAGGTTAGAATT |
| **3209** | TC016342 | tryptophanyl-tRNA synthetase/ligase (Interferon-induced protein 53) | 1.29 |  | GAGGTGTTGAAGCAATTTATGACACCTCGGAAGTTAGCATTTGATGTTTAATTTGAAGTG |
|  |  |  |  |  | GCAGGGCGAAAATTACTGATGAGGTGTTGAAGCAATTTATGACACCTCGGAAGTTAGCAT |
| **3746** | TC012575 CG11529 | serine protease | -2.56 | -3.04 | ATCGCAAGCTTTATAAGTGCTAACGGATGCGAAAGTTTAGATCCAACTGGGTACACAAGG |
|  |  |  |  |  | ATCCAACTGGGTACACAAGGATTGATTCTTACAACGGATGGATTAAAAATGCTACAACTT |
| **3906** | TC003872 Fbp2 | alcohol dehydrogenase | -1.44 | -1.39 | GCTAAAAGTGGAACTGTATGGGTGGCCGAAAATGGGCAAGATCCTTACGAATTTATCCTA |
|  |  |  |  |  | TACCACCCAGGGAGAGTTTCGCGCCAAAATAATTTGTAAAAAATGTTATAGTAGGTATAT |
| **3936** | TC012876 Ntf-2 | nuclear transport factor-2 | 1.27 |  | CTGTAGACTCACAACCTATGTTTGATGGGGGAGTACTAATTAATGTCTTAGGTAGATTAC |
|  |  |  |  |  | GATTAATAGAATAATTACGGCTGTAGACTCACAACCTATGTTTGATGGGGGAGTACTAAT |
| **4024** | XM_968699 | na | -2.12 |  | AACGAAGAAAGGCTCTCGAGCGAGAGCGGAAGGAGAGGTTGGACCGTCTGAAGGACGAGA |
|  |  |  |  |  | TTTAACGAGCTGGAGGCGCAGAACAAGCGCCACGACTTCTTGCAGTCATGTCGCGAACAG |
| **4188** | TC015224 | serpin peptidase inhibitor 31 | -1.86 |  | TCTTTTACATCAAGATTAACGATGTCATCCTCTTCGAAGGCAGAGTTTTGGTACCGGAAA |
|  |  |  |  |  | GTCTTTTACATCAAGATTAACGATGTCATCCTCTTCGAAGGCAGAGTTTTGGTACCGGAA |
| **4230** | AB205184 Sr-CI | melanin-inhibiting protein | -1.98 |  | TGACGTGGAGTACTACGTCTACGGAGTCAAAAAAGAAGCCAATAAGTAATACCACCGAAC |
|  |  |  |  |  | ACGGAGTCAAAAAAGAAGCCAATAAGTAATACCACCGAACGTGCTTTAATTGTTGTCTTA |
| **4230** | AB205184 Sr-CI | melanin-inhibiting protein | 1.82 | -1.13 | TGACGTGGAGTACTACGTCTACGGAGTCAAAAAAGAAGCCAATAAGTAATACCACCGAAC |
|  |  |  |  |  | ACGGAGTCAAAAAAGAAGCCAATAAGTAATACCACCGAACGTGCTTTAATTGTTGTCTTA |
| **5757** | TC006775 | histone-binding protein RBBP4 (retinoblastoma-binding protein 4) | -3.19 |  | AGAGTACAAGATCTGGAAGAAAAACACGCCGTTTCTGTATGATCTGGTGATGACACACGC |
|  |  |  |  |  | TACAAGATCTGGAAGAAAAACACGCCGTTTCTGTATGATCTGGTGATGACACACGCGCTA |
| **5818** | TC000069 Pros26 | proteasome | -1.23 | -1.52 | CAAGGATGGAATACAAGAAGACAGCTTCCAGCTGAGAAAGGATTAAACTGTAATTGTTTT |
|  |  |  |  |  | GCTGCAGAAAGAGATATATTTACAGGTGACAGCATTTTGATTAATGTGATTACCAAGGAT |
| **6467** | TC013555 | transcription initiation factor | -1.24 |  | GAGAGTATACCTAAAGATCATCGATTAGATGTGTCAAAAGTTACCCAACAAACTTTAGGA |
|  |  |  |  |  | GTTACCCAACAAACTTTAGGAGTTTTTTCACATGTGATTCCTTCACAAACAGATGCTATT |
| **6626** | XM_317046 | mitochondrial ribosomal protein L24 | 1.24 |  | CAAATCAAGTGGCTCTTGTTGATCCTTCTGACCTGCAAGCAACTTCAGTTGAGTGGAGAT |
|  |  |  |  |  | TATGCAAATGGAAAAAACCTCTCTTGGTAACAAATCAAGTGGCTCTTGTTGATCCTTCTG |
| **6914** | XM_961851 | inhibitor of NFkappaB kinase | -1.56 |  | TCTGAGAGACAAAGAGAACGATGGGAGAACGAGGTGAACATCATGCAAACTCTCAAATGC |
|  |  |  |  |  | TTCTGAGAGACAAAGAGAACGATGGGAGAACGAGGTGAACATCATGCAAACTCTCAAATG |
| **7075** | TC008409 | translation machinery associated protein mct-1 | -1.27 |  | CTTTGTCCACTGATGAACATAGCGAAAATCAACAAGGGAATAGGTGTTGAAAACTGTCAT |
|  |  |  |  |  | TAGGTGTTGAAAACTGTCATTACTTAAATGATGGCTTGTGGCAAATGAAACCTGTTAAGT |
| **7267** | TC007858 | Protein of unknown function (DUF3421) | 1.71 |  | ACGAAAATGGTAAAGTTCTGAATATTGGTCGAGGCTCTTATCAAGAAGAAGTAATTGTGG |
|  |  |  |  |  | CTATTGGTAAACATTTGGTCATTGGCGGTTACGAAAATGGTAAAGTTCTGAATATTGGTC |
| **7267** | TC007858 | Protein of unknown function (DUF3421) | 1.47 |  | ACGAAAATGGTAAAGTTCTGAATATTGGTCGAGGCTCTTATCAAGAAGAAGTAATTGTGG |
|  |  |  |  |  | CTATTGGTAAACATTTGGTCATTGGCGGTTACGAAAATGGTAAAGTTCTGAATATTGGTC |
| **7506** | TC014024 | TLD, TBC domain | -2.17 |  | AAATGCAAATAATGCCTTCGGTGCTGGAAGAAGTGGCTGTCGAACAATCGAACTCTTACA |
|  |  |  |  |  | GAAATGCAAATAATGCCTTCGGTGCTGGAAGAAGTGGCTGTCGAACAATCGAACTCTTAC |
| **7638** | TC009981 CG9471 | NADPH dehydrogenase | -1.69 | -1.39 | AACCAGAGCATCATGGCAAAGTGTGTGGAATCAGCACTAAGCATTAATAATTAATTGTTA |
|  |  |  |  |  | ATGGGACCATGGGGAAAGAACATTTAGATTAAGAAGTGATACCAGATTATGACAATTACA |
| **8122** | XM_001811882 GA12600 | B-cell receptor-associated protein 31-like | -1.51 | -1.48 | AGGTGAAATTGCCCAAAACGATAGTAACGAAGCGCATGATAAAGAGGTAACTGATCTGAA |
|  |  |  |  |  | TTTTGTCATTAGTCATCCGAAGGCTAGTTATTCTCATCTCGTCGCAAGCTGCTCTGTTGG |
| **8689** | XM_965528 | vacuolar ATP synthase | 1.5 |  | AAATTGACGCTAAAGCTGAAGAAGAATTCAACATTGAAAAAGGCCGTCTTGTCCAACAAC |
|  |  |  |  |  | ATGGCTTTCATTGAACAAGAAGCCAATGAAAAAGCCGAAGAAATTGACGCTAAAGCTGAA |
| **9375** | AY337517 Cp1, TC009365 | cathepsin L | 2.82 | 1.09 | CGTTCCAACTTTGTAAATAAATTCTTCACAAACGCAATGTACAAACTGATGAATGCGCAA |
|  |  |  |  |  | GCGTTCCAACTTTGTAAATAAATTCTTCACAAACGCAATGTACAAACTGATGAATGCGCA |
| **9624** | TC008958 CG8331 | receptor expression enhancing protein | -1.15 | 1.23 | CTGTATAGAAGATTGGTGCGTCCATACTTTTTGAAACATCACAACAATGTGGATGAAATG |
|  |  |  |  |  | CTGTATAGAAGATTGGTGCGTCCATACTTTTTGAAACATCACAACAATGTGGATGAAATG |
| **9828** | XM_969848 CG17347 | dynactin | 1.29 |  | ACAAAGTAACAGTTACTAATGGCTGTATTATTGGTGCAGGTTGTAAAGTTACACAGGAAC |
|  |  |  |  |  | CAAAGTAACAGTTACTAATGGCTGTATTATTGGTGCAGGTTGTAAAGTTACACAGGAACC |
| **10177** | TC009539 CG11738 | lethal (1) G0004; RNA-binding | 1.27 |  | CGCCTTGGCGCTGATGAGACTGGATGACTTATTTATAGAAACTTTTGAAATTAAAGATGT |
|  |  |  |  |  | GCCTTGGCGCTGATGAGACTGGATGACTTATTTATAGAAACTTTTGAAATTAAAGATGTA |
| **10290** | TC006245 | suppressor of Ty 4 homolog 1; transcription elongation factor SPT4 | 1.21 |  | AGACACAAGTCAACGATAGTGCGCCTCAATCTCAATTTTTATACATTTAACTTCACTGAT |
|  |  |  |  |  | CTAATTTTGATGGAATGATTGCTGCTATGAGTCCTGATGACAGTTGGGTTTGTAAATGGC |
| **11225** | TC012531 | aminoacylase | -1.35 |  | GACTTCCAGCTATTGGATTTTCTCCGATTAATAACACTCCAGTATTGCTACATGATCACG |
|  |  |  |  |  | TGGATCGCTTTTAAAGAAGCCACTGACAAGCTCGGCCTTAAGTTGAAACCGCAGATATTT |
| **11374** | TC011289 | GTPase subunit SAR (Secretion associated, Ras-related) | -1.5 |  | GACTTCCAGCTATTGGATTTTCTCCGATTAATAACACTCCAGTATTGCTACATGATCACG |
|  |  |  |  |  | TGGATCGCTTTTAAAGAAGCCACTGACAAGCTCGGCCTTAAGTTGAAACCGCAGATATTT |
| **11938** | TC015577 | Pleiohomeotic (Pho) | -3.05 |  | GTTGCAATAAAAATGTTCCGTGACAATTCAGCGATGCGAAAGCACCTACACACACACGGA |
|  |  |  |  |  | TTGCAATAAAAATGTTCCGTGACAATTCAGCGATGCGAAAGCACCTACACACACACGGAC |
| **12173** | XM_962191 | esterase | -2.57 |  | AGGATCACGCAGTACAGAGTTCAAAAAATCTGGACAGATTTTGCGAAATATTTAAACCCT |
|  |  |  |  |  | ATATATTTGGCGAATGTACGACGACTACTATGACAACTCCGACTTGGCAATTTTTCCGAT |
| **12201** | TC010448 | 6-phosphogluconate dehydrogenase | -1.41 |  | CTTTTGATAAAGACCCTAATCTTCGTTCTTTGCTCCTGGCTCCTTTCTTCCTTGATGCCA |
|  |  |  |  |  | TTCTTGGGTCAGATAAAGGCGGCTTTTGATAAAGACCCTAATCTTCGTTCTTTGCTCCTG |
| **12482** | TC008729 | thiolase; acetyl transferase | -1.34 |  | AAATTGCCTACTCTCTTTAAGGAAAATGGATTAGTCACTGCAGGCTCAGCATCTGGAATT |
|  |  |  |  |  | AATTGCCTACTCTCTTTAAGGAAAATGGATTAGTCACTGCAGGCTCAGCATCTGGAATTT |
| **12590** | TC016344 CG4367 | chitin-binding | 9.88 |  | GATGTGGTCCTCAAGAAACATTCCGATCATGTTCAGATATAACAATTTTGTAAAATGGGC |
|  |  |  |  |  | GGCAGTACCACTGTAAATCTTACTGTCAAGTTACCTGATGGTTTGACTTGTGAAAGATGT |
| **12590** | TC016344 CG4367 | chitin-binding | 24.6 | 1.62 | GATGTGGTCCTCAAGAAACATTCCGATCATGTTCAGATATAACAATTTTGTAAAATGGGC |
|  |  |  |  |  | GGCAGTACCACTGTAAATCTTACTGTCAAGTTACCTGATGGTTTGACTTGTGAAAGATGT |
| **12894** | TC011564 | thaumatin | 5.8 | 1 | AATGTTCCTACCCCATTTTCAACGACTGTCCGGACGAGTTGAAGCTGTTCAATAAGGATG |
|  |  |  |  |  | GAATGTTCCTACCCCATTTTCAACGACTGTCCGGACGAGTTGAAGCTGTTCAATAAGGAT |
| **12948** | XM_970914 | na | -1.21 | 1 | CAAAAAGGCGTTTACGCGAATCAGAGGACTACGCTAGCATCTTGTTGAAAACTAAGGACC |
|  |  |  |  |  | TGTGTTTTTGGGTTGGTTACAAGTCATTTTTGAGCCCTTGGCGCGCAAAAAGGCGTTTAC |
| **13233** | XM_963136 | xanthine dehydrogenase | -2.88 |  | ACCCTTTCATGATGAAGTATAAAGTCGCGTTTGATGATCAGGGTAAAATTTTGGGAATTC |
|  |  |  |  |  | TATATAACAACTGTGGTTATTCGGTTGATTTGTCACCTTCAAGTTTTAGGAAACGAAGCC |
| **13321** | TC010472 CG10753 | small nuclear ribonucleoprotein at 69D | -1.36 |  | AGTTTACCTCTAGAAACACTCCTGATTGATGATACACCTAAAGCAAAGGCTAAGAAGAAG |
|  |  |  |  |  | CGATATTATATTTTGCCAGATAGTTTACCTCTAGAAACACTCCTGATTGATGATACACCT |
| **13451** | XM_964186 CG15117 | glycosyl hydrolase | 2.11 |  | GCCTTTTTACGAGGAACCGACAACCTAAGTCCAGCGCTTATCTCATGAGAAAACGTTACT |
|  |  |  |  |  | GCTTATCTCATGAGAAAACGTTACTGGGCCTTGGCACAAAGCTTGGACGATACTGATGTT |
| **14493** | TC010829 betaTub56D | beta tubulin | 1.57 |  | AGCTTCTGGGAGCGTCGCCTACAGAAAACTGAGCGTCAAAGATTTGACAGAACAAATGTT |
|  |  |  |  |  | CGACTTTCATTGCCAACACTACATCTATTCAAGAACTGTTCAAGCGCATACAAGAACAGT |
| **14493** | TC010829 betaTub56D | beta tubulin | 1.4 |  | AGCTTCTGGGAGCGTCGCCTACAGAAAACTGAGCGTCAAAGATTTGACAGAACAAATGTT |
|  |  |  |  |  | CGACTTTCATTGCCAACACTACATCTATTCAAGAACTGTTCAAGCGCATACAAGAACAGT |
| **15484** | TC004597 | synaptic vesicle protein | -1.72 |  | AAGTGATATTATAGACAATAGCAAACAGCTTTTCGTATCTCCAATCCTCAAAATTCACCG |
|  |  |  |  |  | GCAAACAGCTTTTCGTATCTCCAATCCTCAAAATTCACCGATCATTTCTATTACCATCAA |
| **15790** | AB021700 | 86 kDa early-staged encapsulation inducing protein | -2.88 |  | ACCTCTTACTATGAAGTGTACCAGAAGGTAATGGGTGTGAGTGACCAACTAATCGCCAAC |
|  |  |  |  |  | CAGGAACTCTCGTCAAATCTACAATTACATGCACGACAGGACCTCTTACTATGAAGTGTA |
| **16145** | AY325895 | chitinase | -2.66 |  | ACAAGAGATGAGATCTGCATTCGACAGTGCTGGATATATATTCCATTGCGGTACCTGGAA |
|  |  |  |  |  | AGCAACTTTGTAAAACTCTTACAAGAGATGAGATCTGCATTCGACAGTGCTGGATATATA |
| **16243** | TC004032 CG14949 | na | 2.02 |  | AAAGGGTCCGAGATCATCTCCAACCTACCCAAGCTCCTGGGGCTCACGAAGAAACAGGAC |
|  |  |  |  |  | GAAAGGGTCCGAGATCATCTCCAACCTACCCAAGCTCCTGGGGCTCACGAAGAAACAGGA |
| **16243** | TC004032 CG14949 | na | 3.96 | 2 | AAAGGGTCCGAGATCATCTCCAACCTACCCAAGCTCCTGGGGCTCACGAAGAAACAGGAC |
|  |  |  |  |  | GAAAGGGTCCGAGATCATCTCCAACCTACCCAAGCTCCTGGGGCTCACGAAGAAACAGGA |
| **16411** | XM_963397 CG4367 | chitin-binding | -5.68 | -1.49 | AGAATACTGGTAAATTTGGGCAAGGAAAAATTGTTCGAACTTACAAAGCTGGAAGCGTTA |
|  |  |  |  |  | CTTATACAGATGCTCACCCTCAAGACAACGAGAATACTGGTAAATTTGGGCAAGGAAAAA |
| **16635** | XM_002523507 | serine-threonine protein kinase | -1.54 |  | AAATGCTGCAGGTGGAAATTTGGAAGAAGCTGTCAGTGGTATTAATTCAGAGTTTAAAGA |
|  |  |  |  |  | AATGCTGCAGGTGGAAATTTGGAAGAAGCTGTCAGTGGTATTAATTCAGAGTTTAAAGAG |
| **16751** | TC013662 CG15918 | chitin deacetylase 6 | 2.02 |  | ACTCTTGACTACTTGTCAACTCAAGAATGTCGTACGGGTACAACTTGTCCGTCAGAGTCA |
|  |  |  |  |  | TCTGGATTGCTCCAATAAACATCTACCAAAGGAAATAACAGCATAGAGTGTAATTCTTCA |
| **16751** | TC013662 CG15918 | chitin deacetylase 6 | 1.68 | 1.39 | ACTCTTGACTACTTGTCAACTCAAGAATGTCGTACGGGTACAACTTGTCCGTCAGAGTCA |
|  |  |  |  |  | TCTGGATTGCTCCAATAAACATCTACCAAAGGAAATAACAGCATAGAGTGTAATTCTTCA |
| **16973** | DQ356032^c^ TC013672 | serine protease | 2.3 | 2.46 | GAGAACGACATTGGTCTTATCAGGATAGATGCTGCTTAATAAAACCAACGATCACATACG |
|  |  |  |  |  | GGAGAACGACATTGGTCTTATCAGGATAGATGCTGCTTAATAAAACCAACGATCACATAC |
| **17333** | XM_961998 | juvenile hormone-inducible protein | 1.23 |  | GTCCTACTTCCTCTTCACTTGTCTGTCTGAGGATGATATCCCAAATTTTGATGAAATTGT |
|  |  |  |  |  | TGTCCTACTTCCTCTTCACTTGTCTGTCTGAGGATGATATCCCAAATTTTGATGAAATTG |
| **17333** | XM_961998 | juvenile hormone-inducible protein | 1.43 |  | GTCCTACTTCCTCTTCACTTGTCTGTCTGAGGATGATATCCCAAATTTTGATGAAATTGT |
|  |  |  |  |  | TGTCCTACTTCCTCTTCACTTGTCTGTCTGAGGATGATATCCCAAATTTTGATGAAATTG |
| **17985** | TC006158 | na | -1.22 |  | ACTCTGATTATCACCTACCATGGAGAACTCGACTTGACAAATGGCAATCTCGGAGACTTC |
|  |  |  |  |  | TCTACCTGACATGCACACCGGTTTCAATTACACTCTGATTATCACCTACCATGGAGAACT |
| **18326** | AY337517 Cp1, TC009365 | cathepsin L-like protein | 1.94 |  | ACAAAACGGCGGAATCGACTCAGAAGGCGCACTACCCCTACGAAATGGCGGACGGAATTG |
|  |  |  |  |  | CACAAAACGGCGGAATCGACTCAGAAGGCGCACTACCCCTACGAAATGGCGGACGGAATT |
| **18373** | XM_966328 CG6843 | IP14452p, transcription initiation factor | 1.26 |  | CAACACAGGCACAACAAATTATGACTATATACCTAATGATGAAAATGCTGCTGTGAGTTT |
|  |  |  |  |  | CAGATGAGAGAAGATGGATTAGCTATTAAAAAACAGTATCTGAACAACACAGGCACAACA |
| **18492** | DQ356032^c^ TC013672 | serine protease | 2.04 | 2 | ATCATCCATCAGGATATACTAGAACTGCCGCTTATCGCGACTGGATAGACAGTGTAATTG |
|  |  |  |  |  | TCATCCATCAGGATATACTAGAACTGCCGCTTATCGCGACTGGATAGACAGTGTAATTGC |
| **18860** | AY327800^c^ CG10477 | cockroach allergen-like protein | 8.37 |  | GCTGGGGTGAACCAAAGAAATTCTAATCTACACTATTTGGTTTGTAAATAGTATGTGCTT |
|  |  |  |  |  | GGCTGGGGTGAACCAAAGAAATTCTAATCTACACTATTTGGTTTGTAAATAGTATGTGCT |
| **18860** | AY327800^c^ CG10477 | cockroach allergen-like protein | 7.42 | 2.92 | GCTGGGGTGAACCAAAGAAATTCTAATCTACACTATTTGGTTTGTAAATAGTATGTGCTT |
|  |  |  |  |  | GGCTGGGGTGAACCAAAGAAATTCTAATCTACACTATTTGGTTTGTAAATAGTATGTGCT |
| **19513** | TC004948 | peroxiredoxin | -1.82 |  | GTCAACGATATCAAATCATACTGTCGTGACATTCCAGGCGATTTTCCGTACCCCATCATC |
|  |  |  |  |  | TCAACGATATCAAATCATACTGTCGTGACATTCCAGGCGATTTTCCGTACCCCATCATCG |
| **19654** | TC010829^d^ betaTub97EF | beta tubulin | 1.53 |  | GTAGACTATGTTATGTATGACACCCAGACCAAGAACTCGTCGTATTTCGTCGAGTGGATC |
|  |  |  |  |  | TAGACTATGTTATGTATGACACCCAGACCAAGAACTCGTCGTATTTCGTCGAGTGGATCC |
| **19654** | TC010829 betaTub97EF | beta tubulin | 1.38 |  | GTAGACTATGTTATGTATGACACCCAGACCAAGAACTCGTCGTATTTCGTCGAGTGGATC |
|  |  |  |  |  | TAGACTATGTTATGTATGACACCCAGACCAAGAACTCGTCGTATTTCGTCGAGTGGATCC |
| **19654** | TC010829 betaTub97EF | beta tubulin | 1.35 | 1.39 | GTAGACTATGTTATGTATGACACCCAGACCAAGAACTCGTCGTATTTCGTCGAGTGGATC |
|  |  |  |  |  | TAGACTATGTTATGTATGACACCCAGACCAAGAACTCGTCGTATTTCGTCGAGTGGATCC |
| **19676** | DQ356028^c^, TC016372 | serine protease homolog | 2.15 |  | CTTACCTCTGCCGTGTGTATTTATGGATACGACACTTTCACCGTTCTGGTAGGAGTGATA |
|  |  |  |  |  | TTATGGATACGACACTTTCACCGTTCTGGTAGGAGTGATAGATTTGAATGGCTCTGGTGT |
| **19702** | TC005795 CG1244 | na | 1.62 | -1.32 | AGAATGGTAAGACTGGTTTTGTCTTCGACGAAGAAGACTTTGCTGCCCAGTACACGTTCA |
|  |  |  |  |  | CAAGGTCTTCACCGGGATCAAGAATGGTAAGACTGGTTTTGTCTTCGACGAAGAAGACTT |
| **20445** | TC007958 | na | 2.74 |  | TACGAAGAGTTCTGGATTGACGGATTCAACTTTGAAAACGATCTACGATCAGGAGAAAGA |
|  |  |  |  |  | TTTGAAAACGATCTACGATCAGGAGAAAGAGATTGTTATCTGCGGTCAAGCCGATGAGGA |
| **20445** | TC007958 | na | 4.58 | 1.39 | TACGAAGAGTTCTGGATTGACGGATTCAACTTTGAAAACGATCTACGATCAGGAGAAAGA |
|  |  |  |  |  | TTTGAAAACGATCTACGATCAGGAGAAAGAGATTGTTATCTGCGGTCAAGCCGATGAGGA |
| **20501** | TC007958 | na | 1.28 | 1 | TACGAAGAGTTCTGGATTGACGGATTCAACTTTGAAAACGATCTACGATCAGGAGAAAGA |
|  |  |  |  |  | TTTGAAAACGATCTACGATCAGGAGAAAGAGATTGTTATCTGCGGTCAAGCCGATGAGGA |
| **20629** | AF312017^c^ CG9701 | β-glucosidase | 3.58 | 2 | TTACAATAACATTATCACAACTTAGACACGTCGATTGGGACTACTACCCAGACTGGCCGC |
|  |  |  |  |  | TTTACAATAACATTATCACAACTTAGACACGTCGATTGGGACTACTACCCAGACTGGCCG |
| **20794** | TC016344 CG4367 | chitin-binding | 3.86 | 1 | AGAATACTGGTAAATTTGGGCAAGGAAAAATTGTTCGAACTTACAAAGCTGGAAGCGTTA |
|  |  |  |  |  | CTTATACAGATGCTCACCCTCAAGACAACGAGAATACTGGTAAATTTGGGCAAGGAAAAA |
| **20870** | TC013187 | Rho family small GTP binding protein cdc42 | 1.46 |  | CTAGAACCTCCAGAGCCAATTAAACGTAAAAAGTGTGTTATCTTGTAAACAGTAATCCAG |
|  |  |  |  |  | TAGAACCTCCAGAGCCAATTAAACGTAAAAAGTGTGTTATCTTGTAAACAGTAATCCAGC |
| **21620** | TC001310 | allergen Aca s 13 | -2.4 | 2 | TGGAACCAGAGTGTACAAATTCTCCGACACCGAACTTGTTGTCACCATGACCAGTGATAA |
|  |  |  |  |  | TTGGAACCAGAGTGTACAAATTCTCCGACACCGAACTTGTTGTCACCATGACCAGTGATA |
| **21756** | TC012005 CG12099 | RING-finger protein 10 | 1.3 |  | AGTGTGAACACCGAAAATGAACATGCAGGACCGTCTTTTGCTAAAATGTTGGCAAGTGAA |
|  |  |  |  |  | CAGTGTGAACACCGAAAATGAACATGCAGGACCGTCTTTTGCTAAAATGTTGGCAAGTGA |
| **21958** | TC013754 | renin receptor-like protein | -1.13 |  | ACCCAAAAAGTCTAGTGTTCAAAGGCCATGATCATGTGAAAGAAAGCATTTTAAAAGAAG |
|  |  |  |  |  | CCCAAAAAGTCTAGTGTTCAAAGGCCATGATCATGTGAAAGAAAGCATTTTAAAAGAAGT |
| **21958** | TC013754 | renin receptor-like protein | -1.16 |  | ACCCAAAAAGTCTAGTGTTCAAAGGCCATGATCATGTGAAAGAAAGCATTTTAAAAGAAG |
|  |  |  |  |  | CCCAAAAAGTCTAGTGTTCAAAGGCCATGATCATGTGAAAGAAAGCATTTTAAAAGAAGT |
| **23242** | TC014564 | adenosylhomocysteinase | -1.61 |  | CTGTTTGCACATGACGATCCAAACGGCGGTCCTGATCGAAACCTTGAAAGAGCTAGGCCG |
|  |  |  |  |  | GCTGTTTGCACATGACGATCCAAACGGCGGTCCTGATCGAAACCTTGAAAGAGCTAGGCC |

^a^BLAST hits are from TBLASTX of contigs with NCBI nr, filtering <e^-0.05^, including the *D. melanogaster* or *T. castaneum*ortholog when available, with the predicted function based on sequence homology when available; na-no associated sequence and/or function. ^b^Microarray fold difference is the relative expression in Cry3Aa-intoxicated larvae compared to control.

^c^RNA-Seq fold difference is relative fold increase based on the number of reads from pyrosequencing of larvae exposed to Cry3Aa for 24 h compared to unexposed control larvae.
