# Supplementary material for: Transcriptome Profiling of the Intoxication Response of Tenebrio molitor Larvae to Bacillus thuringiensis Cry3Aa Protoxin
Source: PLoS One. 2012 Apr 25;7(4):e34624. doi: 10.1371/journal.pone.0034624 (PMC3338813; doi:10.1371/journal.pone.0034624)
Supplement: Table S7 — Primers used in qPCR to compare the expression of two CBD3 transcripts in Cry3Aa-intoxicated Tenebrio molitor larvae compared to control. (DOCX) [file pone.0034624.s009.docx]

**Table S7.**

| **Primer Name** | **Sequence** | **Predicted Function of Target** |
| --- | --- | --- |
| Contig_12590_Forward | TACCATCGTCACACAGTCC | Chitin-binding protein 3 |
| Contig_12590_Reverse | GTCGTCAACATATACTCTGCC |  |
| Contig_16411_Forward | CTTCACTTTCATTAGCAACCAC | Chitin-binding protein 3 |
| Contig_16411_Reverse | CCCTCAAGACAACGAGAATAC |  |
| NORM_Contig_18688_Forward | TGCTTCAACTGCCTTGTTC | Ribosomal protein L24 |
| NORM_Contig_18688_Reverse | ACGTGTTATTTCTGCTCGTC |  |
